# MCP-1/CCL2 in Fungal Infections: Balancing Immune Recruitment and Immunopathology

**DOI:** 10.3390/jof12100732

**Published:** 2026-10-01

**Authors:** Xinyi (Silverdew) Shi, Erin E. McClelland

**Affiliations:** Department of Biomedical Sciences, Marian University Wood College of Osteopathic Medicine, Indianapolis, IN 46222, USA; xshi333@marian.edu

**Keywords:** MCP-1, CCL2, CCR2, fungal infections, antifungal immunity, monocyte recruitment, immunopathology

## Abstract

Fungal infections cause substantial morbidity and mortality, particularly in immunocompromised individuals, yet the immune mechanisms that distinguish protective inflammation from tissue-damaging responses remain incompletely understood. Monocyte chemoattractant protein-1, also known as C-C motif chemokine ligand 2 (MCP-1/CCL2), is frequently induced during fungal infection and regulates the recruitment of CCR2-expressing monocytes and other immune-cell populations. This review synthesizes experimental and clinical evidence concerning MCP-1/CCL2 across infections caused by *Cryptococcus*, *Candida*, *Aspergillus*, *Pneumocystis*, dimorphic fungi, Mucorales, environmental molds, and other fungal species. The available literature shows that MCP-1/CCL2 is neither uniformly protective nor pathogenic. Early and localized production often supports antifungal defense by recruiting functionally competent immune cells, particularly in the lung and at some mucosal surfaces. In contrast, delayed or persistent expression may accompany uncontrolled fungal growth, ineffective chemotaxis, immune reconstitution, and tissue injury, especially in the central nervous system, kidney, cornea, and chronically inflamed airways. Its biological and clinical significance therefore depends on the infected tissue, timing of expression, host immune status, fungal traits, CCR2 function, and the identity and competence of recruited cells. MCP-1/CCL2 is unlikely to serve as a stand-alone biomarker or universal therapeutic target but may be useful in stage-specific therapeutic strategies and biomarker panels.

## 1. Introduction

Fungal infections are an increasingly important global health concern, ranging from superficial mucosal or skin disease to invasive infections that affect multiple organs and tissues, including the lungs, bloodstream, brain, kidney, and cornea. Recent estimates suggest that invasive fungal diseases affect more than 6.5 million people and contribute to approximately 3.8 million deaths each year [1], while the World Health Organization has identified major fungal pathogens as global priorities for research, surveillance, and treatment development [2]. Antifungal resistance is also increasing worldwide, making it more difficult to treat infections caused by pathogens, such as *Candida*, *Aspergillus*, *Cryptococcus*, and other clinically important fungi [3]. As fungal infections become more common and more difficult to treat, it is increasingly important to understand how the immune system controls fungal growth without causing excessive tissue injury.

Monocyte chemoattractant protein-1 (MCP-1), also known as C-C motif chemokine ligand 2 (CCL2), is an inflammatory chemokine closely associated with monocyte and macrophage recruitment during tissue inflammation [4]. MCP-1/CCL2 signals mainly through its receptor C-C chemokine receptor type 2 (CCR2) and promotes inflammatory monocyte trafficking from bone marrow to inflamed tissues [5,6]. Because CCR2 also responds to other chemokine ligands, CCR2-dependent phenotypes should not automatically be attributed specifically to MCP-1/CCL2. In some fungal infection models, MCP-1/CCL2-directed recruitment brings antifungal cells to infected tissue and improves fungal control [7,8,9]; in others, the same pathway sustains inflammation and contributes to tissue injury [10,11,12]. Thus, the effect of MCP-1/CCL2 depends on when and where it is produced, whether responding cells retain functional CCR2 signaling, and whether those cells improve fungal control or amplify tissue injury.

Evidence is organized by fungal species and evaluated according to infection site, timing, host immune status, CCR2 function, and the identity and antifungal competence of recruited cells. This approach distinguishes conditions in which MCP-1/CCL2-mediated recruitment improves fungal control from those in which it accompanies ineffective or tissue-damaging inflammation.

## 2. Literature Search Strategy

A targeted literature search was conducted using PubMed, Google Scholar, Google, and ResearchGate to identify studies examining MCP-1/CCL2 in fungal infections. The final search was performed on 15 July 2026, and no lower publication-date limit was imposed. Search terms included combinations of “MCP-1,” “CCL2,” “monocyte chemoattractant protein-1,” “fungal infection,” “fungal disease,” and specific fungal genera or species discussed in this review. Reference lists of relevant articles were reviewed to identify additional studies.

Original studies were included when they examined MCP-1/CCL2 expression, regulation, genetic variation, functional manipulation, receptor-associated recruitment, or clinical associations in a fungal infection or fungal-exposure model. Studies were excluded when they did not contain MCP-1/CCL2-related data or were unrelated to fungal disease. Studies reporting unchanged or non-significant MCP-1/CCL2 responses were retained when they provided informative contrasts or helped define the limits of MCP-1/CCL2 interpretation.

To distinguish the strength of evidence supporting an MCP-1/CCL2-specific biological role, studies summarized in Supplementary Tables S1–S5 were categorized as strong, moderate, or weak. Strong evidence included direct manipulation of MCP-1/CCL2 with measurement of functional outcomes, such as fungal burden, cellular recruitment, pathology, or survival. Moderate evidence included experimental manipulation of CCR2, an upstream host pathway, a fungal factor, or a cellular process that linked MCP-1/CCL2 to a relevant biological response without directly establishing MCP-1/CCL2-specific causality. Weak evidence was primarily associative or descriptive, including changes in MCP-1/CCL2 mRNA or protein levels, biomarker associations, or treatment-associated changes without direct demonstration of MCP-1/CCL2-dependent function. These categories reflect the strength of evidence for an MCP-1/CCL2-specific role rather than overall study quality.

## 3. Fungal Species

### 3.1. Pulmonary Cryptococcosis

Pulmonary cryptococcosis provides some of the strongest evidence that MCP-1/CCL2 supports antifungal leukocyte recruitment. Direct manipulation demonstrates protective recruitment of monocytes, macrophages, T cells, natural killer (NK) cells, and NKT cells, while receptor and host-pathway studies show why chemokine production alone is insufficient when downstream recruitment fails. A summary of these studies is provided in Supplementary Table S1.

#### 3.1.1. Direct Functional Evidence Supports a Protective MCP-1/CCL2 Recruitment Axis

The strongest support for a protective role of MCP-1/CCL2 comes from experiments in which the chemokine itself is disrupted. During pulmonary *Cryptococcus neoformans* infection, MCP-1/CCL2 protein levels increase in bronchoalveolar lavage fluid (BALF) in parallel with monocyte/macrophage and cluster of differentiation 4-positive (CD4^+^) T-cell recruitment. Neutralization of MCP-1/CCL2 markedly reduces recruitment of these populations and increases pulmonary fungal burden approximately threefold, directly linking MCP-1/CCL2-dependent recruitment with fungal control [8]. MCP-1/CCL2 also contributes to recruitment of selected innate lymphocyte populations. Genetic MCP-1/CCL2 deficiency reduces pulmonary accumulation of NK and Vα14 NKT cells, whereas γδ T-cell recruitment is preserved, indicating that MCP-1/CCL2 does not regulate all recruited populations equally [13,14].

These effects depend on a functional downstream receptor pathway. CCR2-deficient mice produce normal or increased BALF MCP-1/CCL2 protein levels but nevertheless show defective macrophage and CD8^+^ T-cell recruitment, type 2-skewed inflammation, poor fungal control, and increased dissemination [15]. Importantly, direct comparison of MCP-1/CCL2 neutralization with CCR2 deficiency shows that the two phenotypes are not equivalent. Both reduce macrophage and T-cell recruitment into infected lungs, but CCR2 deficiency additionally disrupts antigen-presenting cell (APC) accumulation and T helper 1 (Th1) priming in lung-associated lymph nodes [16]. Consistent with this broader receptor function, MCP-1/CCL2, CCL7, and CCL12 messenger RNA (mRNA) are induced together during pulmonary infection, while comparison of CCR2^+/+^ and CCR2^−/−^ mice demonstrates that CCR2 is required for conventional dendritic-cell recruitment and formation of bronchovascular mononuclear infiltrates [17]. Thus, MCP-1/CCL2 is an important component of pulmonary recruitment, but CCR2-dependent antifungal immunity also reflects signaling by additional ligands.

Supporting models further separate MCP-1/CCL2 abundance from protection. Elevated levels of MCP-1/CCL2 occur in selected B-cell and Fc receptor deficiencies [18], while neutrophil depletion alters disease outcome without changing pulmonary MCP-1/CCL2 protein levels [19]. Thus, the protective effect identified by direct MCP-1/CCL2 manipulation depends on intact downstream immunity.

#### 3.1.2. Timing and Cellular Source Shape the Pulmonary MCP-1/CCL2 Response

The timing of MCP-1/CCL2 production is also important. Early pulmonary MCP-1/CCL2 expression often accompanies effective cellular recruitment, whereas delayed or persistent production may not compensate for an immune response that has become ineffective. Interleukin (IL)-12 treatment increases pulmonary MCP-1/CCL2 mRNA and protein levels through an interferon (IFN)-γ-dependent pathway, with early induction accompanying macrophage and CD4^+^ T-cell accumulation across two pulmonary models [20,21]. By contrast, transient tumor necrosis factor (TNF)-α blockade suppresses early MCP-1/CCL2, IL-12, and IFN-γ production. BALF MCP-1/CCL2 protein levels later recover, but fungal clearance does not, indicating that delayed restoration does not replace an effective early response [22].

During persistent pulmonary cryptococcosis, MCP-1/CCL2 protein levels peak during early and subacute infection and decline during chronic disease, although levels remain above baseline. Pulmonary macrophages and epithelioid cells within granulomas are major local sources, and macrophage phagocytosis of opsonized *C. neoformans* stimulates MCP-1/CCL2 production ex vivo [23]. Tissue and time dependence are also evident in 5-lipoxygenase (5-LO)-deficient mice, which show increased lung MCP-1/CCL2 protein levels early after *Cryptococcus deneoformans* infection but lower brain MCP-1/CCL2 protein levels later in infection, emphasizing that regulation of this chemokine changes across compartments and disease stages [24].

The cellular source of MCP-1/CCL2 also differs within the respiratory compartment, which may determine where the chemokine gradient is amplified. Human blood monocytes produce substantially more MCP-1/CCL2 mRNA and secreted protein than resident bronchoalveolar macrophages after cryptococcal exposure [25], while mast cells release MCP-1/CCL2 protein through spleen tyrosine kinase (Syk)-associated signaling and generate supernatants that promote monocyte migration [26].

Airway epithelial cells show the opposite pattern. Neither *C. neoformans* nor live *C. gattii* strongly induces MCP-1/CCL2 protein alone, and both may suppress MCP-1/CCL2 induced by allergen or cytokine stimulation under selected exposure conditions [27,28]. These in vitro findings suggest that strong myeloid MCP-1/CCL2 production does not extend uniformly across the respiratory compartment.

#### 3.1.3. Fungal and Host Factors Regulate MCP-1/CCL2 Within Broader Inflammatory Networks

Fungal traits alter the magnitude of pulmonary MCP-1/CCL2 induction. Melanization and laccase activity increase MCP-1/CCL2 protein levels [29], while fungal strain and inoculum also modify the response [30]. However, greater chemokine production does not consistently improve fungal control, separating induction from protective function.

Host signaling studies identify several routes that regulate pulmonary MCP-1/CCL2 production. Macrophage receptor with collagenous structure (MARCO) deficiency reduces early pulmonary MCP-1/CCL2 mRNA and protein levels [31], while matrix metalloproteinase (MMP) inhibition reduces MCP-1/CCL2 mRNA together with macrophage and neutrophil recruitment [32]. Sphingosine kinase 1/sphingosine-1-phosphate (SK1/S1P) signaling supports macrophage MCP-1/CCL2 production during granuloma-forming infection [33], while IL-4 receptor alpha deficiency reduces MCP-1/CCL2 mRNA together with alveolar macrophage and dendritic-cell recruitment [34]. Additional in vitro models show that toll-like receptor 9 (TLR9) stimulation increases macrophage MCP-1/CCL2 production [35], while *Pleurotus pulmonarius* crude extract increases MCP-1/CCL2 transcription in human THP-1 monocytes and conditioned macrophages [36]. These findings broaden the range of stimuli associated with MCP-1/CCL2 induction but do not establish a chemokine-specific functional effect.

Overall, pulmonary cryptococcosis establishes a clear hierarchy of evidence. Direct MCP-1/CCL2 neutralization or deletion demonstrates protective recruitment of selected mononuclear and lymphoid populations, while CCR2 studies show that the receptor has broader functions mediated by additional ligands. Upstream signaling, fungal traits, and cellular source modify MCP-1/CCL2 production but do not by themselves establish a chemokine-specific role. Protection therefore requires early local MCP-1/CCL2 production, functional CCR2-dependent recruitment, and recruited cells capable of fungal control. This pulmonary pattern provides an important contrast with central nervous system (CNS) cryptococcosis in the upcoming section.

### 3.2. CNS Cryptococcosis and Human Cryptococcal Meningitis

Compared with pulmonary cryptococcosis, CNS disease presents a different setting for MCP-1/CCL2-mediated recruitment. In the lung, early recruitment often supports fungal control, whereas in the CNS, the same inflammatory process carries a greater risk of tissue injury. Experimental studies most consistently link sustained MCP-1/CCL2-associated signaling with inflammatory monocyte entry, intracranial inflammation, and neuropathology. However, this relationship is not uniformly pathologic. MCP-1/CCL2 expression also appears within protective Th1-associated responses under select conditions. Human studies add another layer of complexity by shifting the evidence from mechanism toward inflammatory phenotype, immune reconstitution inflammatory syndrome (IRIS) risk, and prognosis. A summary of these studies is provided in Supplementary Table S2.

#### 3.2.1. Sustained MCP-1/CCL2-Associated Recruitment Contributes to CNS Inflammation and Neuropathology

The strongest experimental evidence for a pathologic role of MCP-1/CCL2 in cryptococcal CNS disease comes from models in which inflammatory recruitment is directly linked to neurologic injury. During *C. neoformans* meningoencephalitis, brain MCP-1/CCL2 protein levels increase during the later inflammatory phase of infection and peak near the onset of mortality. CCR2 signaling promotes inflammatory monocyte entry into the brain and contributes to neuropathology, indicating that the consequences of this recruitment differ substantially from the predominantly protective pattern observed in pulmonary disease [11]. Because CCR2 responds to multiple ligands, this phenotype reflects a broader CCR2-dependent recruitment pathway rather than an MCP-1/CCL2-specific effect alone.

Direct manipulation of MCP-1/CCL2 provides strong evidence that the chemokine itself contributes to disease under select conditions. Microglia isolated from restraint-stressed mice produce detectable MCP-1/CCL2 protein, whereas control microglia show little or no detectable protein. Addition of recombinant MCP-1/CCL2 increases brain fungal burden, while anti-MCP-1/CCL2 treatment reduces fungal burden in stressed mice, directly linking stress-associated MCP-1/CCL2 production with worsened cerebral cryptococcosis [37]. These findings suggest that local MCP-1/CCL2 production becomes detrimental when it arises within an already dysregulated CNS inflammatory state.

Later-stage inflammation also depends on upstream host pathways. In 5-LO-deficient mice infected with *C. deneoformans*, brain MCP-1/CCL2 protein levels are reduced during late disease together with decreased leukocyte infiltration, while early pulmonary MCP-1/CCL2 production shows the opposite pattern. This contrast reinforces that MCP-1/CCL2 regulation depends on both tissue compartment and infection stage [24]. IL-23 provides another upstream signal. IL-23p19-deficient mice show reduced brain MCP-1/CCL2 mRNA and protein levels during *C. neoformans* infection, placing MCP-1/CCL2 within an IL-23-associated inflammatory pathway in the infected CNS [38].

Fungal phenotype also shapes the magnitude and consequence of the CNS response. Rats infected intracisternally with a mucoid variant of *C. neoformans* show substantially higher brain MCP-1/CCL2 protein levels than those of animals infected with the smooth parent strain. Higher MCP-1/CCL2 production accompanies greater monocyte/macrophage infiltration, increased cryptococcal polysaccharide accumulation, elevated intracranial pressure, and shorter survival [39]. In this setting, stronger MCP-1/CCL2-associated recruitment does not improve fungal control and instead occurs within a more damaging inflammatory phenotype. Together, these studies support a model in which sustained or dysregulated MCP-1/CCL2-associated recruitment becomes increasingly harmful in the inflammation-sensitive CNS.

#### 3.2.2. MCP-1/CCL2 Expression Is Not Inherently Pathologic and Depends on the Surrounding Immune Response

Although several CNS models associate MCP-1/CCL2 production with neuropathology, levels of the chemokine do not uniformly indicate harmful inflammation. In mice immunized with *C. neoformans* culture filtrate antigen and subsequently challenged intracerebrally, MCP-1/CCL2 mRNA and protein levels increase as part of a broader Th1-associated response that includes TNF-α, IFN-γ, and multiple additional chemokines. This coordinated inflammatory response is associated with improved fungal clearance, suggesting that MCP-1/CCL2 participates differently when recruitment occurs within an effective cell-mediated immune program [40].

Compartment-specific studies further demonstrate why MCP-1/CCL2 concentration alone is difficult to interpret. In TNF/lymphotoxin (LT)-α-deficient mice with disseminated cryptococcosis, MCP-1/CCL2 protein levels are higher in plasma and lung, lower in spleen, and unchanged in brain compared to those in wild-type (WT) mice, yet the deficient animals remain markedly more susceptible to infection [41]. Thus, increased MCP-1/CCL2 levels in one compartment do not compensate for failure of the broader inflammatory network, and similar concentrations in the brain do not imply equivalent host protection.

These studies separate MCP-1/CCL2 abundance from outcome. Protective CNS responses include MCP-1/CCL2 production within coordinated Th1 immunity, whereas persistent MCP-1/CCL2-associated recruitment during poorly controlled inflammation accompanies monocyte accumulation and neurologic injury.

#### 3.2.3. Human MCP-1/CCL2 Protein Levels Reflect CNS Inflammatory Phenotype and IRIS Risk

Human cryptococcal meningitis studies largely support MCP-1/CCL2 as a marker of inflammatory state rather than direct evidence of causality. The clearest clinical pattern concerns cryptococcosis-associated immune reconstitution inflammatory syndrome (C-IRIS). In patients with human immunodeficiency virus (HIV)-associated cryptococcal meningitis, higher baseline CSF MCP-1/CCL2 protein levels are associated with subsequent development of C-IRIS [42]. A separate prospective cohort similarly shows that CSF MCP-1/CCL2 concentrations exceed plasma concentrations at initiation of combination antiretroviral therapy (cART), supporting compartmentalized CNS chemokine production. A higher CSF MCP-1/CCL2 to C-X-C motif chemokine ligand (CXCL)10 ratio is also associated with later C-IRIS, suggesting relatively stronger myeloid-cell chemotactic signaling in patients predisposed to inflammatory deterioration [43].

This relationship extends beyond HIV-associated disease. In non-HIV cryptococcal meningitis, higher baseline CSF MCP-1/CCL2 protein levels are also associated with later IRIS, and MCP-1/CCL2 protein levels are linked to leukotriene A4 hydrolase (LTA4H) polymorphisms in the same cohort [44]. These findings support elevated CSF MCP-1/CCL2 protein levels as part of an inflammatory state that predisposes to immune reconstitution-associated complications. They do not establish that MCP-1/CCL2 production alone drives IRIS, particularly because the clinical measurements are observational and occur within broader cytokine and leukotriene responses.

Higher MCP-1/CCL2 protein levels do not uniformly indicate more severe neurologic disease. Patients with relatively higher CD4^+^ T-cell counts [45] or altered mental status [46] may have lower CSF MCP-1/CCL2 protein levels despite other evidence of substantial inflammation or neurologic disease. Thus, MCP-1/CCL2 reflects a particular inflammatory phenotype rather than a linear severity scale.

Comparative and outcome cohorts reinforce this limitation. CSF MCP-1/CCL2 production differs from some control or alternative meningitis groups [47,48] but overlaps with other CNS opportunistic infections [49], while CSF and plasma measurements show different short-term [50] and long-term [51] prognostic associations. These findings support compartment-specific interpretation rather than a universal MCP-1/CCL2 threshold.

Overall, CNS cryptococcosis separates MCP-1/CCL2-mediated recruitment from a uniformly protective role. Direct MCP-1/CCL2 manipulation supports a pathologic effect under stress-associated immune dysregulation, while CCR2, 5-LO, IL-23, and fungal variant studies identify broader pathways associated with CNS recruitment and injury. Human studies are primarily associative and support CSF MCP-1/CCL2 levels as part of an inflammatory and IRIS-risk profile rather than a stand-alone severity marker. Interpretation therefore requires information on the measured compartment, immune state, and accompanying inflammatory response.

### 3.3. Candida

*Candida* studies separate three steps in MCP-1/CCL2 biology: chemokine induction, cellular recruitment, and antifungal function after recruitment. Direct functional studies support a protective role when this sequence remains intact, whereas several susceptibility models demonstrate that high MCP-1/CCL2 production becomes disconnected from protection when recruitment or cellular function fails. A summary of these studies is provided in Supplementary Table S3.

#### 3.3.1. MCP-1/CCL2 Induction Is Shaped by Tissue, Fungal Phenotype, and Upstream Signaling

Experimental vaginal candidiasis provides both an early example of local MCP-1/CCL2 induction and direct evidence that the response is biologically relevant. Following intravaginal *Candida albicans* infection, MCP-1/CCL2 protein levels increase in vaginal tissue by day 2 and remain elevated during persistent infection, while MCP-1/CCL2 mRNA increases from day 4 onward. In contrast, MCP-1/CCL2 protein levels do not increase in draining lymph nodes. Direct MCP-1/CCL2 neutralization increases vaginal fungal burden, demonstrating that a spatially restricted MCP-1/CCL2 response contributes to early fungal control [9].

MCP-1/CCL2 production is not restricted to leukocytes. Oral and vaginal epithelial cells secrete MCP-1/CCL2 protein after *C. albicans* exposure [52], infected gingiva shows increased MCP-1/CCL2 mRNA with local protein staining [53], and gastric tissue shows increased MCP-1/CCL2 mRNA [54]. Human osteoblasts release MCP-1/CCL2 protein after *C. glabrata* exposure [55], a *Candida*-responsive dental pulp stem-cell population shows increased MCP-1/CCL2 transcription [56], and fungal particles induce MCP-1/CCL2 mRNA in adipocytes [57]. These descriptive studies broaden the range of tissue cells capable of contributing to a local MCP-1/CCL2 signal.

The magnitude of this response also depends on how the fungus is recognized. During oropharyngeal candidiasis (OPC), loss of the shared IL-12/IL-23 p40 subunit prevents the normal transient rise in oral MCP-1/CCL2 mRNA, placing MCP-1/CCL2 downstream of a p40-dependent inflammatory pathway [58]. Fungal properties further modify chemokine induction. *C. albicans* mannosylation mutants produce distinct kidney and spleen MCP-1/CCL2 protein responses [59], invasive strains induce greater endothelial MCP-1/CCL2 protein production than those of an invasion-deficient rim101 mutant [60], and loss of ECE1 or candidalysin reduces early renal MCP-1/CCL2 protein levels during systemic infection [61]. Thus, cell-wall structure, invasive capacity, and secreted virulence factors each influence the host MCP-1/CCL2 response.

The *C. albicans* water-soluble extract (CAWS) arteritis model provides a more detailed mechanism linking fungal recognition to chemokine production. Dectin-2 sensing of fungal mannans induces early MCP-1/CCL2 production by cardiac tissue-resident macrophages, followed by CCR2-dependent monocyte recruitment. Recruited cells then promote IL-1β production, which stimulates a later endothelial MCP-1/CCL2 response and amplifies vascular inflammation. Disruption of Dectin-2-associated signaling reduces MCP-1/CCL2 production and protects against arteritis [62]. Although CAWS does not represent live candidiasis, this model demonstrates how fungal carbohydrate recognition initiates and amplifies an MCP-1/CCL2-associated recruitment circuit.

Host regulatory pathways may also suppress or exaggerate MCP-1/CCL2 production. During macrophage exposure to *C. albicans*, candidalysin-associated Galectin-9/T-cell immunoglobulin and mucin domain (Tim)-3 signaling suppresses MCP-1/CCL2 transcription along with broader inflammatory responses, while pathway blockade restores this response [63]. At the opposite extreme, T cells from a patient with a Signal transducer and activator of transcription 1 (STAT1) gain-of-function mutation and recurrent mucocutaneous candidiasis show enhanced interferon-induced MCP-1/CCL2 transcription, although the single-patient design and absence of infected tissue measurements limit interpretation [64].

#### 3.3.2. Protection Requires an Effective Response to the MCP-1/CCL2 Signal

Once MCP-1/CCL2 is produced, protection depends on whether appropriate immune cells respond to the chemotactic signal. The vaginal neutralization study demonstrates this principle at a mucosal surface, where direct MCP-1/CCL2 neutralization increases fungal burden [9]. The strongest systemic evidence comes from the Tripartite motif-containing protein 72 (Trim72) model of lethal *C. albicans* infection. Trim72 deficiency reduces renal MCP-1/CCL2 protein levels and macrophage accumulation, while recombinant Trim72 increases both. More importantly, pharmacologic MCP-1/CCL2 inhibition reduces macrophage recruitment, increases fungal burden and renal injury, and worsens survival despite Trim72 treatment. Macrophage depletion similarly eliminates Trim72-mediated protection. In both murine and human macrophages, Trim72 also increases MCP-1/CCL2 production and migration through nuclear factor kappa-light-chain-enhancer of activated B cells (NF-κB) and extracellular signal-regulated kinase 1/2 (ERK1/2) signaling [65]. These findings directly establish that MCP-1/CCL2-dependent macrophage recruitment contributes to fungal control.

Other studies define the production and responder sides of the pathway. Type I IFN signaling supports renal MCP-1/CCL2 mRNA expression and cellular MCP-1/CCL2 protein production together with inflammatory monocyte recruitment [10], while LY86 knockdown reduces CCR2 expression and migration toward MCP-1/CCL2 production [66]. Weaker supporting studies show that reduced macrophage MCP-1/CCL2 production accompanies hyperglycemia-associated dysfunction [67] and *Candida*-associated denture stomatitis [68], whereas *C. dubliniensis* immunization increases bone-marrow MCP-1/CCL2 protein levels during myeloid training [69]. These associations support altered MCP-1/CCL2 production during immune dysfunction or training but do not establish an MCP-1/CCL2-specific mechanism.

Together, these findings support a model in which MCP-1/CCL2-mediated protection requires more than chemokine production. The signal must reach cells with adequate CCR2 responsiveness, and those recruited cells must retain sufficient antifungal function to improve fungal control.

#### 3.3.3. MCP-1/CCL2 Production Becomes Uncoupled from Protection When Recruitment or Fungal Control Fails

The clearest evidence for this uncoupling comes from genetically susceptible mice. SM/J mice with systemic candidiasis have higher serum MCP-1/CCL2 protein levels than those of resistant C57BL/6J mice, yet they recruit fewer inflammatory monocytes to infected kidneys and carry greater fungal burden. Serum MCP-1/CCL2 protein levels also correlate positively with renal fungal burden in mice carrying the susceptibility-associated Carg5 locus [70]. Thus, an abundant circulating chemokine signal does not provide protection when effective cellular recruitment fails.

The same dissociation appears in other susceptibility models, where higher MCP-1/CCL2 L protein levels accompany infection with more virulent *C. albicans* strains [12], C5 deficiency [71], or myeloid protein tyrosine phosphatase 1B (PTP1B) deficiency despite greater fungal burden or inflammation [72].

The reverse relationship also occurs. Macrophage-specific IL-1 receptor antagonist deletion improves fungal clearance while lowering renal MCP-1/CCL2 mRNA, consistent with resolution of fungal-driven inflammation [73]. Other models show why the identity and function of recruited cells matter as much as the chemokine signal itself. Depletion of protective CD169-positive renal resident macrophages worsens candidiasis despite preserved renal MCP-1/CCL2 mRNA and early inflammatory-monocyte and neutrophil recruitment [74]. In the cornea, athymic mice show reduced MCP-1/CCL2 mRNA and leukocyte recruitment during experimental keratitis, linking MCP-1/CCL2-associated recruitment with local inflammatory disease rather than demonstrating a protective effect [75]. Together, these studies show that MCP-1/CCL2-associated recruitment must be interpreted according to which cells enter the tissue and whether their activity improves fungal control or contributes to local injury.

#### 3.3.4. Therapeutic Modulation Reflects the Part of the MCP-1/CCL2 Response Being Corrected

Therapeutic studies further show that MCP-1/CCL2 follows the immune process being altered rather than moving in one direction with clinical improvement. Innate defense regulator (IDR)-1018 [76], sodium new houttuyfonate (SNH) [77], and deoxyribonuclease I (DNase I) plus antimicrobial photodynamic therapy (aPDT) [78] increase MCP-1/CCL2 protein levels during broader treatment-associated immune activation. Because MCP-1/CCL2 is not directly shown to mediate these benefits, these studies are best interpreted as treatment-associated immune activation rather than evidence for MCP-1/CCL2 as the therapeutic mechanism.

Other interventions reduce MCP-1/CCL2 levels as fungal burden or tissue inflammation resolves. Cis-2-dodecenoic acid (BDSF) lowers vaginal MCP-1/CCL2 mRNA [79], while itraconazole and microRNA (miR)-124 reduce both renal MCP-1/CCL2 mRNA and protein levels during improvement of candidiasis-associated acute kidney injury (AKI) [80]. Adenoviral MCP-1/CCL2 delivery [81] and hinokitiol treatment [82] further demonstrate that the pathway is experimentally modifiable but add little evidence regarding therapeutic causality.

Overall, across *Candida* models, direct neutralization and inhibition studies support a protective role when MCP-1/CCL2 recruits functional antifungal cells. The broader literature explains why chemokine abundance alone is insufficient: CCR2-responsive cells must reach infected tissue and retain useful antifungal activity. When recruitment fails, protective resident cells are lost, or fungal-driven inflammation persists, high MCP-1/CCL2 levels no longer predict protection.

### 3.4. Aspergillus

*Aspergillus* studies show that the effect of MCP-1/CCL2 differs sharply between keratitis, invasive pulmonary disease, and chronic allergic inflammation. The strongest functional studies support a beneficial role for MCP-1/CCL2 when it promotes macrophage antifungal activity or recruits alternative effector cells. In contrast, persistent expression after fungal control fails or during chronic allergic disease more often accompanies ongoing inflammation. A summary of these studies is provided in Supplementary Table S4.

#### 3.4.1. Aspergillus Keratitis: Fungal Sensing, Macrophage Function, and Treatment-Associated Inflammation

Fungal keratitis provides one of the strongest mechanistic examples of MCP-1/CCL2 function outside the lung. In infected mouse corneas, inhibition of indoleamine 2,3-dioxygenase (IDO) increases corneal MCP-1/CCL2 and CCR2 mRNA and protein levels. Direct MCP-1/CCL2 neutralization or CCR2 blockade reduces macrophage phagocytosis and fungal killing, whereas addition of recombinant MCP-1/CCL2 enhances both responses. MCP-1/CCL2-CCR2 signaling also shifts macrophages toward an M2-like phenotype in this model, indicating that polarization state alone does not predict antifungal competence [83]. These findings provide direct evidence that MCP-1/CCL2 regulates macrophage antifungal function rather than serving only as a recruitment marker.

Upstream fungal-recognition studies support this local response. In rat corneal epithelium, *A. fumigatus* infection progressively increases MCP-1/CCL2 mRNA, while Dectin-1 blockade with laminarin reduces the early response, indicating partial dependence on Dectin-1 signaling [84]. A separate rat keratitis model similarly demonstrates increased corneal MCP-1/CCL2 mRNA and epithelial protein localization, although MCP-1/CCL2 levels are not closely correlated with macrophage-inducible C-type lectin (Mincle), suggesting that additional recognition pathways contribute to chemokine induction [85].

Several keratitis treatments reduce MCP-1/CCL2 mRNA and/or protein levels together with fungal burden and broader inflammation. Ebselen [86], sanguinarine [87], a β-glucan-specific nanobody [88], pseudolaric acid B [89], and perillaldehyde [90] all lower MCP-1/CCL2 mRNA and/or protein levels while improving one or more measures of fungal burden, inflammatory-cell infiltration, oxidative stress, or disease severity. Because these interventions simultaneously reduce fungal burden and broader inflammatory signaling, declining MCP-1/CCL2 levels are best interpreted as part of disease resolution rather than direct evidence that MCP-1/CCL2 itself is the principal therapeutic target.

Conversely, whole glucan particle vaccination [91] and amphotericin B-based immune modulation [92] increase MCP-1/CCL2 protein levels within broader protective responses, showing that successful intervention does not require chemokine reduction. These two studies suggest that treatment-associated MCP-1/CCL2 changes reflect whether an intervention primarily reduces inflammatory burden or enhances antifungal immune preparedness.

#### 3.4.2. Pulmonary Invasive Aspergillosis: MCP-1/CCL2 Production Supports Compensatory Antifungal Recruitment

The strongest pulmonary evidence supports a protective role for MCP-1/CCL2 during invasive aspergillosis, particularly when neutrophil-mediated defense is impaired. In neutropenic mice infected with *A. fumigatus*, lung MCP-1/CCL2 protein levels increase rapidly during early infection while remaining undetectable in serum. Direct MCP-1/CCL2 neutralization increases mortality and pulmonary fungal burden and selectively reduces early NK-cell recruitment. Adoptive-transfer experiments further demonstrate that CCR2 expression on NK cells supports their migration into infected lungs and contributes to fungal control. Once NK cells are depleted, additional MCP-1/CCL2 neutralization no longer further worsens fungal burden, directly linking the protective effect of MCP-1/CCL2 to NK-cell recruitment [7].

Other models place MCP-1/CCL2 production within compensatory inflammatory networks. TNF neutralization reduces the rapid increase in lung MCP-1/CCL2 protein after challenge with killed hyphae [93], while CCL17 neutralization or CCR4 deficiency increases pulmonary MCP-1/CCL2 protein levels together with improved fungal control [94].

However, later MCP-1/CCL2 elevation does not necessarily restore effective antifungal protection in immunocompromised hosts. In a solid-organ transplant-like model, immunosuppressed mice show reduced early lung MCP-1/CCL2 protein levels followed by increased levels at 48 h, when fungal burden is already high [95]. Chlorine-exposed mice similarly develop increased susceptibility despite higher pulmonary MCP-1/CCL2 protein levels and exaggerated inflammation [96]. In neutropenic rats, MCP-1/CCL2 protein levels increase progressively in infected lung but not serum or contralateral lung, while amphotericin B reduces pulmonary MCP-1/CCL2 protein levels together with fungal-load markers [97]. Together, these studies suggest that once key antifungal defenses are impaired, increased MCP-1/CCL2 production alone is insufficient to compensate for the underlying immune deficit and may instead reflect persistent fungal stimulation and tissue injury.

#### 3.4.3. Allergic and Chronic Airway Aspergillosis: Timing Shifts MCP-1/CCL2 Function from Fungal Clearance to Inflammatory Remodeling

Allergic airway models demonstrate a distinct time-dependent transition in MCP-1/CCL2 function. In nonsensitized mice challenged with live *A. fumigatus* conidia, early MCP-1/CCL2 activity promotes macrophage recruitment and fungal clearance. Early neutralization reduces macrophage accumulation, increases conidial persistence, and accelerates eosinophilic inflammation, airway hyperresponsiveness, and fibrosis. In sensitized mice, adenoviral delivery of MCP-1/CCL2 during early disease improves fungal clearance and reduces airway disease. In contrast, neutralization during established allergic disease reduces lymphocyte recruitment, IL-4 production, airway hyperresponsiveness, and mucus-associated pathology without increasing fungal retention [98]. This study directly demonstrates that MCP-1/CCL2 shifts from an early antifungal mediator to a contributor to later allergic inflammation.

The protective effect also depends on functional CCR2 signaling. Sensitized CCR2-deficient mice challenged with live *A. fumigatus* conidia show elevated pulmonary MCP-1/CCL2 protein levels despite impaired fungal clearance, delayed macrophage recruitment, reduced neutrophil recruitment, and exaggerated allergic inflammation [99]. The importance of this pathway differs when fungal clearance is no longer required. In mice repeatedly exposed to nonviable *Aspergillus* antigen, robust type 2 inflammation, airway hyperreactivity, mucus production, and fibrosis still develop despite genetic loss of MCP-1/CCL2 or CCR2 [100]. Together, these studies suggest that MCP-1/CCL2-CCR2 signaling contributes importantly to cellular recruitment and clearance during live fungal exposure but is not required for the development of antigen-driven allergic airway pathology.

Chronic remodeling is also not fully MCP-1/CCL2-dependent. STAT6 deficiency markedly reduces pulmonary MCP-1/CCL2 protein levels while later airway hyperresponsiveness and fibrosis persist [101], whereas human MCP-1/CCL2 polymorphisms are associated with severe asthma with fungal sensitization (SAFS) without functional validation [102]. Thus, MCP-1/CCL2 may modify susceptibility or severity without being required for chronic remodeling.

Chronic upper-airway studies similarly report increased MCP-1/CCL2 mRNA in canine sinonasal aspergillosis [103] and increased MCP-1/CCL2 protein in human fungal-associated rhinosinusitis, although both remain associative [104].

#### 3.4.4. Human and Translational Models

Human models identify several potential cellular sources. Whole-blood exposure to *A. fumigatus* induces MCP-1/CCL2 protein levels that are not eliminated by monocyte or granulocyte depletion [105], while an aspergillosis-on-chip model shows stronger MCP-1/CCL2 production in the endothelial compartment and in the presence of macrophages [106]. These studies support multicellular production without establishing an MCP-1/CCL2-specific functional effect.

Additional mechanistic studies identify upstream pathways controlling this response. *Aspergillus* protease induces MCP-1/CCL2 mRNA and protein production in human airway epithelial cells, while scavenging mitochondrial reactive oxygen species reduces both, linking fungal protease exposure with oxidative signaling [107]. In human monocytes, heat-inactivated *A. fumigatus* strongly induces MCP-1/CCL2 mRNA, and cyclosporine A markedly suppresses this transcriptional response. The corresponding protein change after *A. fumigatus* stimulation is less clear, so the study most strongly supports calcineurin-associated regulation of MCP-1/CCL2 transcription [108]. Human neutrophils and neutrophil-like cells similarly increase MCP-1/CCL2 mRNA after fungal stimulation, while cyclosporine A and tacrolimus suppress this induction as well as reduce monocyte chemoattraction [109]. Together, these studies suggest that calcineurin inhibition may suppress innate chemokine production in addition to its established effects on adaptive immunity.

Clinical evidence is associative. Elevated baseline serum MCP-1/CCL2 protein levels predict later invasive fungal disease in high-risk hematology patients [110], while MCP-1/CCL2 and CCR2 polymorphisms have been associated with invasive aspergillosis susceptibility without functional validation [111,112].

Overall, across *Aspergillus* disease, direct functional studies identify three distinct MCP-1/CCL2 effects: macrophage antifungal activity in keratitis, compensatory NK-cell recruitment during neutropenic pulmonary infection, and a stage-dependent shift from early fungal clearance to later allergic inflammation. The remaining studies largely define conditions that modify these effects, including fungal sensing, immune suppression, cellular source, and host susceptibility. Thus, MCP-1/CCL2 is most informative when linked to the function of the recruited or responding cells rather than interpreted from concentration alone.

### 3.5. Other Fungal Species

Although *Candida*, *Cryptococcus*, and *Aspergillus* provide the largest bodies of evidence in this review, studies in other fungal systems further extend two themes established above: MCP-1/CCL2 often functions within broader CCR2-ligand recruitment networks, and elevated chemokine levels do not by themselves distinguish fungal control from inflammatory injury. The strongest studies in this group define upstream pathways controlling MCP-1/CCL2 production or demonstrate its participation in CCR2-dependent monocyte recruitment, whereas several clinical and exposure studies primarily support its value as a marker of inflammatory state. A summary of these studies is provided in Supplementary Table S5.

#### 3.5.1. Pneumocystis Pneumonia: Epithelial MCP-1/CCL2 Production and Immune-Context-Dependent Inflammation

Alveolar epithelial cells are a major early source of MCP-1/CCL2 during *Pneumocystis* exposure. Fungal stimulation induces epithelial mRNA and protein expression through JNK and IκB kinase (IKK) signaling [113], while IL-1R/MyD88 signaling is required for this response and TLR2/TLR4 are dispensable [114]. MyD88 deletion subsequently reveals stage dependence: it reduces early BALF MCP-1/CCL2 protein levels during active pneumonia but is associated with prolonged MCP-1/CCL2 protein elevation during immune-mediated clearance [115].

TNFR bone marrow chimera studies likewise associate high lung MCP-1/CCL2 mRNA and protein levels with macrophage accumulation and respiratory inflammation [116], while immune-reconstitution experiments show little pulmonary MCP-1/CCL2 mRNA despite increasing organism burden until the host inflammatory response is restored [117].

Human studies broadly support this interpretation, although the evidence remains associative. BALF MCP-1/CCL2 levels differ according to the underlying immune condition in patients with pneumocystis pneumonia and correlate with inflammatory markers rather than fungal burden measures alone [118]. In non-acquired immunodeficiency syndrome (AIDS) immunocompromised patients with *Pneumocystis jirovecii* pneumonia (PJP), MCP-1/CCL2 protein levels are elevated in BALF and blood, but its clinical association is stronger when interpreted relative to anti-inflammatory mediators, particularly through the MCP-1/CCL2-to-TGF-β1 ratio [119,120].

Together, these studies show that alveolar epithelial and parenchymal cells initiate MCP-1/CCL2 production, while the magnitude and persistence of that response are determined more by the host inflammatory state than by organism burden alone.

#### 3.5.2. Dimorphic Fungi: MCP-1/CCL2 in CCR2-Dependent Monocyte Recruitment and Granulomatous Immunity

Studies of dimorphic fungi emphasize a different principle: MCP-1/CCL2 often contributes to antifungal immunity as one component of a broader CCR2-ligand network rather than as an independently essential chemokine.

This is demonstrated most clearly in *Histoplasma capsulatum* infection. CCR2-deficient mice develop severe defects in inflammatory monocyte and dendritic-cell recruitment, progressive fungal dissemination, and mortality, whereas MCP-1/CCL2-deficient mice show only a transient increase in fungal burden and survive infection. The difference is explained by ligand redundancy. CCL7 compensates for the absence of MCP-1/CCL2, while combined disruption of CCL7 in MCP-1/CCL2-deficient mice reproduces a more severe phenotype with increased fungal burden and IL-4 transcription [121]. Thus, the protective unit in histoplasmosis is better understood as the broader CCL2/CCL7-CCR2 axis than production of MCP-1/CCL2 alone. Conversely, erythropoietin-treated mice show higher pulmonary MCP-1/CCL2 protein levels together with greater inflammation and mortality, further separating chemokine abundance from protection [122].

Respiratory vaccination with *Blastomyces dermatitidis* provides a mechanistic example of impaired CCR2-ligand responsiveness. *B. dermatitidis* induces pulmonary MMP2 and traps CCR2^+^ Ly6C^hi^ inflammatory monocytes in the bone marrow, reducing their recruitment to the lung and impairing Th1-cell priming. Although recombinant CCL7 is the ligand used for rescue, monocytes from *B. dermatitidis*-vaccinated mice also show reduced migration and calcium flux in response to addition of exogenous MCP-1/CCL2. MMP inhibition or MMP2 deletion restores monocyte recruitment, while transfer of Ly6C^hi^ monocytes restores T-cell priming [123]. The study therefore supports fungal disruption of the broader CCL2/CCL7-CCR2 recruitment pathway, although it does not isolate an MCP-1/CCL2-specific causal effect.

In *Paracoccidioides brasiliensis* infection, IFN-γ promotes early pulmonary MCP-1/CCL2 induction and organized mononuclear recruitment [124], while chemokine levels decline during later chronic pathology [125]. Macrophage studies identify IL-10 [126], TLR4 [127], and MyD88 [128] as regulators of MCP-1/CCL2 production, and susceptible versus resistant mouse strains differ in the magnitude of this response [129]. These studies define upstream control of MCP-1/CCL2 but provide limited evidence for an MCP-1/CCL2-specific functional role. Outside the lung, MCP-1/CCL2 behaves differently: brain MCP-1/CCL2 protein levels rise later during neuroparacoccidioidomycosis [130], serum MCP-1/CCL2 protein levels increase during treatment rather than active paracoccidioidomycosis [131], and basilar-artery MCP-1/CCL2 mRNA increases during experimental coccidioidal meningitis without closely tracking vasculitis severity [132].

These studies support a protective role for CCR2-ligand-dependent monocyte recruitment during pulmonary dimorphic fungal infection, while also demonstrating substantial ligand redundancy and weaker associations outside the lung.

#### 3.5.3. Mucormycosis: Dendritic-Cell-Amplified Barrier Responses and Inflammatory Dysregulation

The limited mucormycosis literature suggests that MCP-1/CCL2 production becomes more prominent when myeloid cells participate in the host response. In a human alveolar epithelial–endothelial Transwell model exposed to *Rhizopus arrhizus*, *Rhizomucor pusillus*, or *Cunninghamella bertholletiae*, epithelial-only conditions produce little MCP-1/CCL2 response. Addition of monocyte-derived dendritic cells markedly increases MCP-1/CCL2 mRNA and protein levels together with other inflammatory mediators while reducing fungal invasion across the barrier [133]. This places MCP-1/CCL2 within a myeloid-amplified barrier response rather than a predominantly epithelial response.

Clinical data from COVID-19-associated mucormycosis demonstrate a broader inflammatory phenotype. Patients with mucormycosis have higher serum MCP-1/CCL2 protein levels than COVID-19 controls, and stimulated patient peripheral blood mononuclear cells (PBMCs) release more MCP-1/CCL2 protein. MCP-1/CCL2 also correlates with several inflammatory cytokines and is higher in participants with diabetes or corticosteroid exposure [134]. These findings support MCP-1/CCL2 as part of systemic inflammatory dysregulation, but the overlapping effects of COVID-19, diabetes, corticosteroid treatment, and fungal disease limit attribution specifically to mucormycosis.

#### 3.5.4. Other Localized and Environmental Fungal Diseases

Environmental mold models show that pulmonary MCP-1/CCL2 induction varies with fungal exposure and host signaling. *Stachybotrys chartarum* induces pulmonary MCP-family chemokines across different strains [135] and toxin-producing phenotypes [136], while *Bjerkandera adusta* provides stronger mechanistic evidence by linking MCP-1/CCL2 production to MyD88 and selected TLR pathways [137].

At other barrier sites, MCP-1/CCL2 responses are similarly variable. *Fusarium solani* keratitis produces sustained local MCP-1/CCL2 protein levels that decline with treatment [138], dermatophyte-exposed keratinocytes show little early MCP-1/CCL2 protein production [139], *Malassezia sympodialis* induces mast-cell MCP-1/CCL2 production [140], and *Pseudogymnoascus destructans*-infected bat wing shows increased MCP-1/CCL2 transcription despite limited leukocyte infiltration [141]. These studies mainly demonstrate tissue-specific inflammatory responses rather than direct MCP-1/CCL2 function.

#### 3.5.5. Model Yeast and Transcriptional Regulation of MCP-1/CCL2

Although *Saccharomyces cerevisiae* is not a major invasive fungal pathogen, it provides one of the clearest molecular models for regulation of MCP-1/CCL2 transcription during fungal recognition. Following intraperitoneal infection, MCP-1/CCL2, CCL7, and CCL12 levels increase rapidly together with recruitment of CCR2-positive inflammatory monocytes. Calcineurin inhibition suppresses these chemokines, reduces monocyte recruitment, and delays fungal clearance. At the transcriptional level, calcineurin-nuclear factor of activated T cells c1 (NFATc1) binds the MCP-1/CCL2 promoter and relieves B-cell lymphoma 6 (BCL6)-mediated repression, while loss of NFATc1 increases BCL6 occupancy and suppresses CCL2, CCL7, and CCL12 expression [142].

This model defines a calcineurin-NFATc1-BCL6 transcriptional mechanism for CCR2-ligand production and complements pathogenic fungal studies that also link calcineurin signaling to MCP-1/CCL2 induction.

Overall, across these less extensively studied fungi, the strongest mechanistic evidence centers on epithelial signaling in *Pneumocystis*, ligand redundancy in dimorphic fungal infection, and transcriptional regulation in the *Saccharomyces* model. Most remaining studies are descriptive and place MCP-1/CCL2 within local or systemic inflammatory responses without establishing a direct functional role. These findings reinforce the distinction between MCP-1/CCL2-specific effects and broader CCR2-ligand or inflammatory phenotypes.

## 4. Discussion

Comparison across fungal diseases suggests that MCP-1/CCL2 is most informative when viewed as one component of a recruitment pathway rather than as an inherently protective or pathogenic mediator. A productive response requires MCP-1/CCL2 to be produced locally at a useful stage of infection, responsive cells to reach the infected tissue through functional CCR2 signaling, and those recruited cells to retain antifungal activity. Disruption at any of these steps helps explain why increased MCP-1/CCL2 protein levels are associated with fungal control in some models but with persistent infection or inflammatory injury in others. Figure 1 summarizes these major outcomes.

### 4.1. Protective MCP-1/CCL2 Responses Require Effective Recruitment and Antifungal Cell Function

The protective role of MCP-1/CCL2 is best understood as a sequence rather than as a direct consequence of chemokine abundance. First, a local MCP-1/CCL2 signal must arise while recruitment is still useful. Direct neutralization or genetic disruption across pulmonary cryptococcosis, candidiasis, and neutropenic aspergillosis consistently demonstrates that loss of this signal reduces recruitment of protective leukocyte populations and worsens fungal control [7,8,9,13,65]. Upstream signaling pathways determine when and where this chemokine signal is generated, but those studies establish regulation rather than MCP-1/CCL2-specific function. The important distinction is therefore between producing MCP-1/CCL2 and successfully converting that signal into antifungal immunity.

The next step is effective CCR2-dependent trafficking. High MCP-1/CCL2 protein levels do not restore protection when responding cells fail to leave the circulation or enter the infected tissue, as illustrated by pulmonary cryptococcosis, systemic candidiasis, and the *Blastomyces* vaccination model [15,70,123]. At the same time, CCR2-dependent recruitment is broader than MCP-1/CCL2 alone. CCR2 deficiency produces more extensive immune defects than MCP-1/CCL2 disruption in pulmonary cryptococcosis [16], whereas in histoplasmosis, CCL7 compensates for MCP-1/CCL2 loss, with combined ligand disruption producing a substantially more severe phenotype [121]. Thus, receptor-based phenotypes establish the importance of the CCR2 recruitment system but should not automatically be interpreted as MCP-1/CCL2-specific effects.

Even successful trafficking is not sufficient if the recruited population lacks the function needed at that site. Loss of protective renal resident macrophages worsens candidiasis despite preserved MCP-1/CCL2 production and early leukocyte recruitment [74], whereas direct MCP-1/CCL2 and CCR2 manipulation in *Aspergillus* keratitis alters macrophage phagocytosis and fungal killing [83]. The broader importance of CCR2-positive myeloid cells is also supported by pulmonary *A. fumigatus* studies outside the MCP-1/CCL2-specific literature: Espinosa et al. demonstrate that CCR2-positive inflammatory monocytes and their monocyte-derived dendritic-cell progeny support neutrophil antifungal activity and directly kill fungal conidia [143]. Together, these findings place cellular function, rather than chemokine concentration, at the end of the protective pathway.

### 4.2. MCP-1/CCL2 Becomes Ineffective or Harmful When Recruitment Is Mistimed, Incomplete, or Excessive

The same sequence explains why increased MCP-1/CCL2 sometimes accompanies poor outcomes. A chemokine response that occurs after a critical protective window has been missed does not necessarily restore immunity. In pulmonary cryptococcosis, recovery of MCP-1/CCL2 production after early TNF-α blockade does not restore fungal clearance [22]. Likewise, in immunocompromised pulmonary aspergillosis, later MCP-1/CCL2 elevation occurs when fungal burden and inflammatory injury are already established [95,96,97]. These findings suggest that MCP-1/CCL2 is most effective before irreversible defects in host defense or uncontrolled fungal growth dominate the response.

A different problem emerges when recruitment itself begins to contribute more to injury than to clearance. Allergic aspergillosis provides the clearest temporal example because early MCP-1/CCL2 production supports fungal clearance, whereas later neutralization reduces established airway inflammation [98]. CNS cryptococcosis demonstrates a similar principle in a tissue with limited tolerance for inflammatory-cell accumulation. CCR2-dependent monocyte recruitment contributes to neuropathology, and direct MCP-1/CCL2 manipulation under stress-associated immune dysregulation alters cerebral fungal burden in the same harmful direction [11,37]. *Pneumocystis* further separates chemokine production from organism burden because MCP-1/CCL2 becomes prominent when host inflammatory responses are restored and may remain elevated during immune-mediated clearance [115,117]. These are not opposing roles of the chemokine so much as different stages at which the cost and benefit of continued recruitment have changed.

The apparently conflicting literature therefore follows a common pattern. MCP-1/CCL2 loses protective value when the response occurs too late, when CCR2-responsive cells fail to reach or function within infected tissue, or when continued recruitment adds inflammatory injury after additional cellular influx provides little antifungal benefit. The biological meaning of an elevated MCP-1/CCL2 measurement therefore depends on where the recruitment sequence has succeeded or failed rather than on chemokine concentration alone. The representative studies and factors that distinguish these outcomes are summarized in Table 1.

### 4.3. Translational Implications for Biomarkers and Therapeutic Targeting

This framework also explains why MCP-1/CCL2 has limited value as an isolated biomarker. A single concentration does not reveal whether the chemokine represents productive recruitment, failed compensation, or ongoing inflammatory injury. In cryptococcal meningitis, higher baseline CSF MCP-1/CCL2 protein levels are associated with subsequent IRIS, yet lower levels also occur in patients with substantial neurologic disease, and the MCP-1/CCL2 to CXCL10 ratio provides information not captured by MCP-1/CCL2 alone [42,43,46]. The same principle appears in PJP, where the MCP-1/CCL2-to-TGF-β1 ratio is more informative than measurement of the chemokine alone, and in aspergillosis and mucormycosis, where circulating MCP-1/CCL2 levels reflect broader host inflammatory states [110,119,120,134]. Compartment is equally important because CSF, BALF, infected tissue, serum, and plasma reflect different parts of the response and should not be interpreted interchangeably [43,51,97,118]. MCP-1/CCL2 is therefore more informative when combined with disease stage, fungal burden, tissue compartment, and other inflammatory markers than when interpreted as a linear measure of severity.

The same reasoning argues against uniform therapeutic augmentation or blockade. Increasing MCP-1/CCL2 levels is most likely to help when inadequate recruitment limits fungal control and the responding population remains functional, whereas reducing the pathway may be beneficial once persistent recruitment contributes substantially to tissue injury. Allergic aspergillosis demonstrates both possibilities within the same disease model, with early augmentation and later neutralization producing different beneficial effects [98]. The opposite effects of MCP-1/CCL2 inhibition in cerebral cryptococcosis and candidiasis reinforce the idea that therapeutic direction depends on disease stage, tissue, and the effector population being recruited rather than on whether MCP-1/CCL2 levels are simply high or low [9,37,65].

### 4.4. Limitations of the Current Evidence and Future Directions

Several limitations restrict how broadly these findings should be interpreted. First, the strength of evidence differs substantially among studies. Only a limited number directly neutralize, delete, supplement, or otherwise manipulate MCP-1/CCL2 while measuring fungal burden, pathology, recruitment, or survival. A larger proportion manipulate CCR2, upstream signaling pathways, fungal traits, immunosuppressive conditions, or treatments and then measure MCP-1/CCL2 levels as one component of the resulting inflammatory response. These studies are valuable for identifying regulation and biological associations, but they do not establish that MCP-1/CCL2 itself causes the accompanying phenotype. The Supplementary evidence tables distinguish these levels of support to reduce overinterpretation.

Second, much of the functional evidence comes from mouse models. These models permit direct chemokine neutralization, genetic deletion, cell transfer, and tissue-specific analysis that are difficult to perform in humans, but the recruited cell populations, fungal inocula, routes of infection, immune deficiencies, and disease timing do not fully reproduce human fungal disease. Murine models therefore provide mechanistic evidence under controlled conditions, but the direction and magnitude of MCP-1/CCL2 effects require confirmation in human tissue models and longitudinal clinical cohorts before therapeutic or biomarker conclusions are drawn.

Third, interpretation of CCR2-dependent phenotypes requires attention to ligand redundancy. *Histoplasma* provides direct evidence that CCL7 compensates for MCP-1/CCL2 deficiency [121], while the *Blastomyces* vaccination model shows that fungal interference with CCR2-ligand responsiveness affects both CCL2- and CCL7-associated recruitment [123]. Future experiments that compare MCP-1/CCL2-specific manipulation with CCR2 disruption and measurement of other CCR2 ligands would better separate ligand-specific effects from broader receptor-dependent recruitment.

Additionally, studies also measure MCP-1/CCL2 in different forms and compartments. CCL2 mRNA indicates transcription but not extracellular protein or a functional chemotactic gradient, while tissue-homogenate protein does not identify the producing cell and circulating levels may reflect systemic rather than local inflammation. BALF and CSF measurements better reflect local activity but still do not establish MCP-1/CCL2-dependent recruitment. Future studies should pair local protein measurements with cellular source, CCR2-positive cell trafficking, fungal burden, and direct functional manipulation at matched disease stages.

Importantly, fungal-derived immunomodulatory compounds also represent another area for future study. Experimental studies reviewed here demonstrate that fungal components may regulate MCP-1/CCL2 through defined host pathways, including Dectin-2-dependent MCP-1/CCL2 production in the *Candida albicans* water-soluble extract model and mitochondrial reactive oxygen species-dependent MCP-1/CCL2 induction by *Aspergillus* protease [62,107]. Broader reviews of polysaccharides from edible and medicinal fungi also describe macrophage and cytokine-modulating activity [147,148], raising the possibility that selected fungal-derived products may influence MCP-1/CCL2-associated immune responses. Direct studies are needed to test their effects on MCP-1/CCL2 production, CCR2-dependent recruitment, and antifungal cell function.

Finally, human studies present an additional limitation because most are observational. Immune suppression, HIV status, corticosteroid exposure, diabetes, antifungal therapy, concomitant infections, and differences in fungal burden all influence inflammatory measurements. Larger longitudinal studies with clearly defined clinical phenotypes and matched tissue or fluid sampling are therefore needed before MCP-1/CCL2 can be used for prognosis or treatment selection. The most informative studies are likely to evaluate MCP-1/CCL2 protein levels together with fungal burden, recruited-cell populations, organ injury, and complementary inflammatory markers rather than seeking a universal MCP-1/CCL2 threshold.

## 5. Conclusions

Across fungal infections, MCP-1/CCL2 is best understood as a regulator of immune-cell recruitment whose effect depends on whether recruitment leads to effective fungal control. When MCP-1/CCL2 is produced at a stage when responsive cells reach the infected tissue and retain antifungal function, it supports host defense. When recruitment is mistimed, ineffective, or persists after its protective value has declined, the same pathway instead contributes to inflammatory injury.

This context dependence has important translational consequences. A change in MCP-1/CCL2 concentration cannot by itself distinguish effective antifungal immunity from failed or harmful inflammation, which limits its usefulness as a stand-alone biomarker or universal therapeutic target. Its greatest value may therefore lie in defining broader inflammatory states, particularly when interpreted together with fungal burden, immune-cell responses, and other biomarkers, and in identifying disease settings in which modulation of MCP-1/CCL2-dependent recruitment could be beneficial.

## Figures and Tables

**Figure 1:**
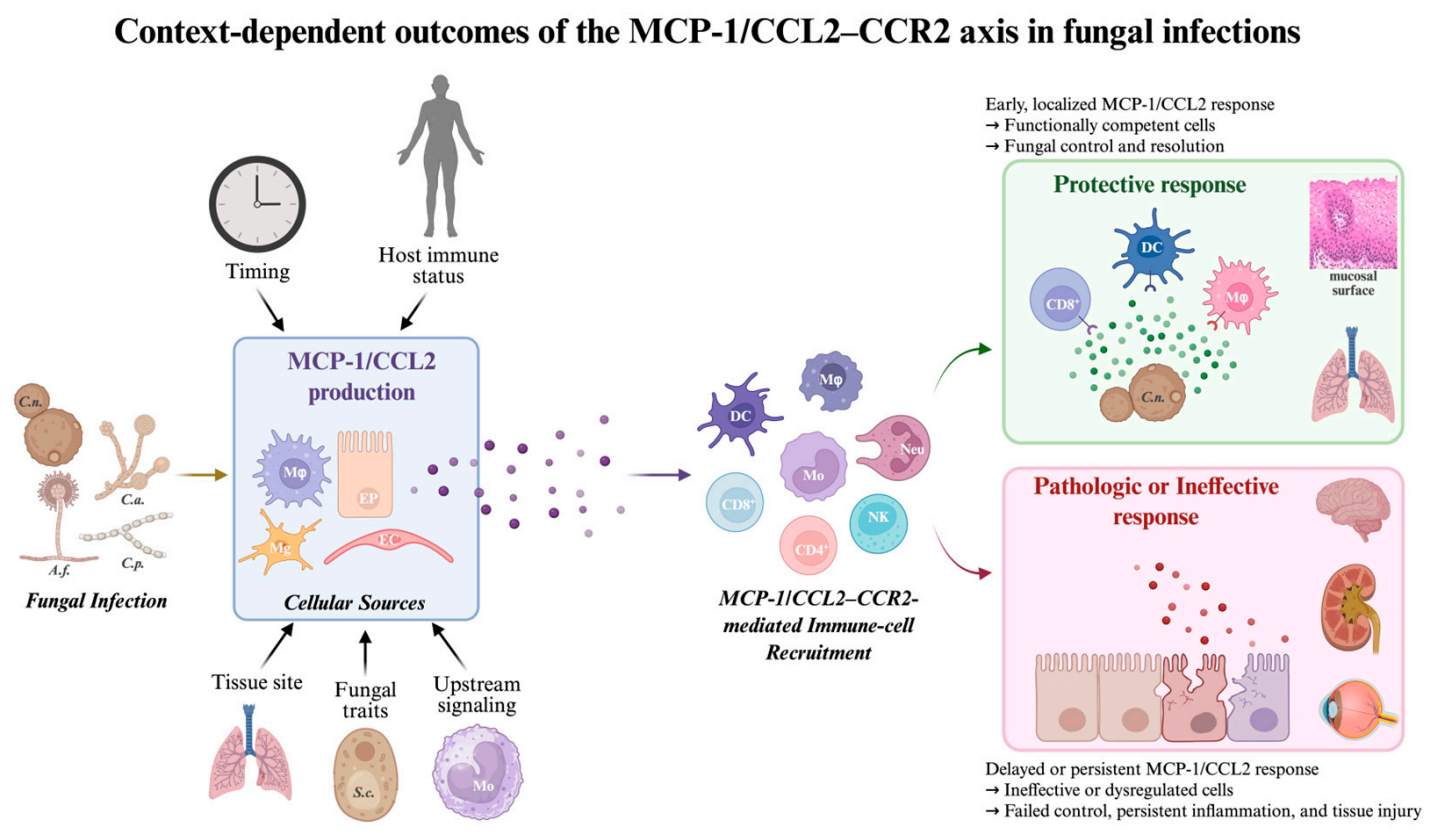
Context-dependent outcomes of the MCP-1/CCL2–CCR2 axis in fungal infections. Fungal infection induces MCP-1/CCL2 production from multiple cellular sources, including myeloid, epithelial, and endothelial cells. The magnitude and timing of this response are influenced by tissue site, host immune status, fungal traits, and upstream signaling pathways. MCP-1/CCL2 contributes to CCR2-mediated recruitment of immune-cell populations, but the biological outcome depends on the timing and localization of the response and the functional competence of recruited cells. Early, localized MCP-1/CCL2 responses support antifungal immunity and resolution when recruited cells effectively control fungal growth. In contrast, delayed or persistent MCP-1/CCL2 responses may accompany ineffective recruitment, dysregulated inflammation, persistent fungal infection, and tissue injury. Representative fungal organisms, cellular sources, recruited immune-cell populations, and affected tissues are shown; these examples are not intended to represent all cell types or fungal diseases discussed in the review. (MCP-1, monocyte chemoattractant protein-1; CCL2, C-C motif chemokine ligand 2; CCR2, C-C chemokine receptor type 2; C.n., *Cryptococcus neoformans*; C.a., *Candida albicans*; A.f., *Aspergillus fumigatus*; C.p., *Coccidioides posadasii*; S.c., *Saccharomyces cerevisiae*; Mφ, macrophage; Mg, microglia; EP, epithelial cell; EC, endothelial cell; DC, dendritic cell; Mo, monocyte; Neu, neutrophil; NK, natural killer cell; CD4^+^, CD4-positive T cell; CD8^+^, CD8-positive T cell.) Created in BioRender. Shi, S. (2026) https://BioRender.com/3bz4x4t, accessed on 21 September 2026.

**Table 1:** Contextual factors determining whether MCP-1/CCL2-mediated recruitment is protective, ineffective, or pathologic.

Contextual Factor	Protective Configuration	Ineffective or Pathologic Configuration	Representative Fungal Examples	Representative Studies
Timing of MCP-1/CCL2 induction	MCP-1/CCL2 is induced early, before extensive fungal growth, allowing timely recruitment of antifungal cells	MCP-1/CCL2 is delayed, persists after fungal control has failed, or remains elevated after its protective value has declined	Early pulmonary cryptococcosis; early neutropenic aspergillosis; late allergic aspergillosis; transplant-associated aspergillosis	[7,8,22,95,98]
Tissue compartment and inflammatory tolerance	Recruitment occurs in tissues that benefit from early mononuclear-cell accumulation, such as the lung or some mucosal surfaces	Recruitment occurs in inflammation-sensitive tissues, where leukocyte accumulation, edema, or tissue injury may outweigh fungal-control benefits	Pulmonary cryptococcosis versus cryptococcal CNS disease; vaginal candidiasis versus renal candidiasis; fungal keratitis	[8,9,10,11,83]
Integrity of CCR2 signaling	MCP-1/CCL2 binds functional CCR2 on responsive leukocytes and supports migration into infected tissue	MCP-1/CCL2 is elevated, but receptor signaling is absent or impaired, so recruitment and fungal control fail	Pulmonary cryptococcosis; allergic aspergillosis	[15,16,99]
Successful tissue entry of responding cells	CCR2-expressing cells leave the circulation and accumulate in the correct infected compartment	MCP-1/CCL2 is abundant systemically, but inflammatory monocytes fail to accumulate effectively in the target organ	Susceptible versus resistant systemic candidiasis models	[70]
Antifungal competence of recruited cells	Recruited macrophages, monocytes, NK cells, or dendritic cells remain capable of phagocytosis, fungal killing, antigen presentation, or cytokine support	Recruited cells are dysfunctional, poorly activated, or unable to replace specialized tissue-resident populations	Trim72-associated renal macrophage recruitment; diabetic candidiasis; renal resident-macrophage depletion; *Aspergillus* keratitis	[65,67,74,83,144]
Identity of the recruited population	MCP-1/CCL2 recruits cell types suited to the immune deficit or tissue, such as macrophages, CD4^+^ T cells, NK cells, or dendritic cells	Recruitment favors cells that amplify inflammation without sufficient fungal control, or the required effector population is not recruited	Pulmonary cryptococcosis; neutropenic aspergillosis; renal candidiasis; cryptococcal CNS disease	[7,8,10,11,93]
Host immune status	MCP-1/CCL2 provides compensatory recruitment when a major defense pathway is missing but alternative effectors remain functional	Immunosuppression delays chemokine induction, metabolic disease weakens macrophage function, or immune restoration produces excessive inflammation	Neutropenic aspergillosis; transplant-associated aspergillosis; diabetic candidiasis; *Pneumocystis* immune reconstitution	[7,67,95,117,145]
Upstream cytokine environment	MCP-1/CCL2 is produced within a coordinated Th1-associated program involving IL-12, IFN-γ, and TNF	MCP-1/CCL2 is induced within inflammatory pathways that promote tissue injury, such as type I IFN, IL-23, 5-LO, or prolonged immune-reconstitution signaling	Pulmonary cryptococcosis; renal candidiasis; cryptococcal CNS disease; *Pneumocystis* pneumonia	[10,20,21,22,24,38,115]
Cellular source of MCP-1/CCL2	Barrier cells or resident immune cells initiate a localized gradient that recruits cells to the appropriate site	Persistent production by microglia, endothelial cells, macrophages, or other local cells maintains damaging inflammation	*Pneumocystis* alveolar epithelium; *Candida* mucosal epithelium; pulmonary cryptococcal monocytes; cerebral cryptococcosis	[25,37,52,113,114]
Fungal virulence traits and phenotype	MCP-1/CCL2 appears as part of an organized containment or vaccine-associated response	Fungal melanin, laccase, candidalysin, altered mannosylation, proteases, or phenotypic switching amplify or redirect MCP-1/CCL2-associated inflammation	Cryptococcal melanin and mucoid switching; *Candida* candidalysin and mannosylation; *Aspergillus* protease; *Coccidioides* vaccination	[29,39,59,61,107,146]
Fungal immune suppression	Host cells produce MCP-1/CCL2 normally in response to fungal or cytokine stimulation	Fungal exposure or fungus-induced host regulatory pathways suppress epithelial or macrophage MCP-1/CCL2 induction.	*Cryptococcus* epithelial immune evasion; *Candida* Tim-3/Galectin-9 signaling	[27,28,63]
CCR2-ligand redundancy	MCP-1/CCL2 cooperates with CCL7, CCL12, and related ligands to maintain recruitment	MCP-1/CCL2 deficiency alone appears mild because other ligands compensate; CCR2 deficiency causes a broader defect	Histoplasmosis; pulmonary cryptococcosis; Blastomyces vaccine responses; *Saccharomyces* model	[16,17,121,123]
Relationship to fungal burden	MCP-1/CCL2 increases early as part of an effective response and then declines as fungal control is achieved	MCP-1/CCL2 increases because fungal burden remains high, tissue invasion progresses, or inflammation fails to resolve	*Coccidioides* vaccination; transplant aspergillosis; renal candidiasis; pulmonary aspergillosis	[12,95,97,142,146]
Disease stage and immune restoration	MCP-1/CCL2 supports clearance during the active antimicrobial phase	The same pathway contributes to injury after immune restoration or during chronic remodeling	*Pneumocystis* IRIS; cryptococcal IRIS; allergic aspergillosis	[42,43,98,117,145]
Treatment context	Increasing MCP-1/CCL2 improves recruitment or macrophage activation when early immunity is inadequate	Decreasing MCP-1/CCL2 is beneficial when treatment reduces fungal burden or established inflammatory injury	Early allergic aspergillosis; Trim72 candidiasis; *Pneumocystis* IRIS; candidiasis-associated AKI; fungal keratitis	[65,80,86,90,98,145]
Biomarker compartment	Local MCP-1/CCL2 in CSF, BALF, or infected tissue reflects compartment-specific immune activation	Serum or plasma MCP-1/CCL2 may not reflect the infected organ and may represent systemic inflammation or failed compensation	Cryptococcal meningitis; pulmonary aspergillosis; *Pneumocystis* pneumonia; systemic candidiasis	[43,70,97,118]
Interpretation with other biomarkers	MCP-1/CCL2 is interpreted with fungal burden, CXCL10, TGF-β1, inflammatory cytokines, and organ-injury markers	MCP-1/CCL2 is interpreted alone as a linear marker of severity	Cryptococcal IRIS; *Pneumocystis* pneumonia; invasive pulmonary aspergillosis	[43,97,119,120]

## Data Availability

No new datasets were generated during the preparation of this review. All information discussed in the manuscript is available in the cited publications.

## Supplementary Materials

The following supporting information can be downloaded at https://www.mdpi.com/article/10.3390/jof12100732/s1. Table S1: MCP-1/CCL2 evidence in pulmonary cryptococcosis; Table S2: MCP-1/CCL2 evidence in CNS cryptococcosis and human cryptococcal meningitis; Table S3: MCP-1/CCL2 evidence in *Candida*; Table S4: MCP-1/CCL2 evidence in *Aspergillus*; Table S5: MCP-1/CCL2 evidence in other fungal species.

## Abbreviations

The following abbreviations are used in this manuscript:

5-LO	5-Lipoxygenase
AFRS	Allergic fungal rhinosinusitis
AHCC	Active hexose correlated compound
AIDS	Acquired immunodeficiency syndrome
AKI	Acute kidney injury
APC	Antigen-presenting cell
aPDT	Antimicrobial photodynamic therapy
BALF	Bronchoalveolar lavage fluid
BCL6	B-cell lymphoma 6
BDSF	Cis-2-dodecenoic acid
BMDMs	Bone marrow-derived macrophages
C-IRIS	Cryptococcosis-associated immune reconstitution inflammatory syndrome
CAM	COVID-19-associated mucormycosis
cART	Combination antiretroviral therapy
CAWS	Candida albicans water-soluble extract
CBA	Cytometric bead array
CCL	C-C motif chemokine ligand
CCR2	C-C chemokine receptor type 2
CD	Cluster of differentiation
CFU	Colony-forming unit
ChIP	Chromatin immunoprecipitation
CNS	Central nervous system
CpG	Cytosine-phosphate-guanosine
CRP	C-reactive protein
CRSwNP	Chronic rhinosinusitis with nasal polyps
CSF	Cerebrospinal fluid
CXCL	C-X-C motif chemokine ligand
DNase I	Deoxyribonuclease I
DTR	Diphtheria toxin receptor
ELISA	Enzyme-linked immunosorbent assay
ERK1/2	Extracellular signal-regulated kinase 1/2 signaling
GCS1	Glucosylceramide synthase 1
GWAS	Genome-wide association study
GXM	Glucuronoxylomannan
HIV	Human immunodeficiency virus
HSCT	Hematopoietic stem cell transplantation
IDO	Indoleamine 2,3-dioxygenase
IDR	Innate defense regulator
IFN	Interferon
IKK	Inhibitor of κB kinase
IL	Interleukin
IRIS	Immune reconstitution inflammatory syndrome
JNK	c-Jun N-terminal kinase
LDH	Lactate dehydrogenase
LOX-1	Lectin-like oxidized low-density lipoprotein receptor-1
LPS	Lipopolysaccharide
LT	Lymphotoxin
LTA4H	Leukotriene A4 hydrolase
MARCO	Macrophage receptor with collagenous structure
MCP-1	Monocyte chemoattractant protein-1
Mincle	Macrophage-inducible C-type lectin
miR	MicroRNA
MMP	Matrix metalloproteinase
mRNA	Messenger RNA
NF-κB	Nuclear factor kappa-light-chain-enhancer of activated B cells
NFAT	Nuclear factor of activated T cells
NK	Natural killer
ODN	Oligodeoxynucleotides
OPC	Oropharyngeal candidiasis
PBMCs	Peripheral blood mononuclear cells
PJP	Pneumocystis jirovecii pneumonia
PTP1B	Protein tyrosine phosphatase 1B
qRT-PCR	Quantitative reverse transcription real-time polymerase chain reaction
RPA	RNase protection assay
S1P	Sphingosine-1-phosphate
SAFS	Severe asthma with fungal sensitization
SCID	Severe combined immunodeficiency
seq	Sequencing
SK1	Sphingosine kinase 1
SNH	Sodium new houttuyfonate
STAT	Signal transducer and activator of transcription
Syk	Spleen tyrosine kinase
Th1	T helper 1
Tim	T-cell immunoglobulin and mucin domain
TLR	Toll-like receptor
TNF(R)	Tumor necrosis factor (receptor)
TRIF	Toll/Interleukin-1 receptor domain (TIR)-domain-containing adapter-inducing interferon-β
Trim72	Tripartite motif-containing protein 72
WT	Wild-type
