# Supplementary material for: MCP-1/CCL2 in Fungal Infections: Balancing Immune Recruitment and Immunopathology
Source: J Fungi (Basel). 2026 Oct 1;12(10):732. doi: 10.3390/jof12100732 (PMC13705938; doi:10.3390/jof12100732)
Supplement: Supplementary file 1 [file jof-12-00732-s001.zip › jof-4532522-supplementary.pdf]

Journal of Fungi

MDPI

Table S1. MCP-1/CCL2 evidence in pulmonary cryptococcosis

Fungal species	Infection site	Experimental model / evidence type	Functional intervention	MCP-1/CCL2 response	Interpretation	Strength of evidence
Cryptococcus neoformans	Lung	CBA/J mice infected intratracheally; BALF MCP-1/CCL2 protein measured by ELISA*; pulmonary leukocytes and fungal burden assessed	Direct anti-MCP-1/CCL2 neutralization	MCP-1/CCL2 protein was undetectable in uninfected BALF, increased by week 1, and remained elevated through weeks 3–5. Neutralization reduced detectable BALF MCP-1/CCL2 by ~80%	Anti-MCP-1/CCL2 markedly reduced monocyte/macrophage and $CD4^+$ T-cell recruitment and increased pulmonary fungal burden ~3-fold, directly supporting a protective recruitment function	Strong [1]
C. neoformans	Lung	WT and MCP-1/CCL2-deficient mice infected intratracheally; pulmonary NK and NKT cells assessed by flow cytometry; lung mRNA and protein measured over time	MCP-1/CCL2 genetic deletion	MCP-1/CCL2 mRNA is detectable by day 1 and peaks around day 3; protein follows a similar early pattern before peak NKT-cell accumulation	MCP-1/CCL2 is required for efficient recruitment of NK, NKT, and Vα14 NKT cells during early pulmonary infection	Strong [2]
C. neoformans	Lung	WT and MCP-1/CCL2-deficient mice infected intratracheally; pulmonary γδ T-cell, NK-cell, and NKT-cell populations assessed	MCP-1/CCL2 genetic deletion	Loss of MCP-1/CCL2 selectively affects recruitment of some innate lymphocyte populations	γδ T-cell recruitment remains intact, whereas NK and NKT-cell accumulation is reduced, indicating cell-type-specific MCP-1/CCL2-dependent trafficking	Strong [3]
C. neoformans	Lung	$CCR2^{+/+}$ and $CCR2^{-/-}$ mice infected intratracheally; BALF MCP-1/CCL2 protein, pulmonary leukocytes, cytokines, and fungal burden assessed	CCR2 genetic deletion	CCR2-deficient mice retain normal or elevated BALF MCP-1/CCL2 protein levels despite impaired macrophage and $CD8^+$ T-cell recruitment, poor fungal control, and increased dissemination	Elevated MCP-1/CCL2 is insufficient for protection when CCR2 signaling is disrupted; CCR2 deficiency impairs cellular recruitment, promotes type 2 inflammation, and worsens fungal control	Moderate [4]

C. neoformans	Lung and lung-associated lymph nodes	WT mice treated with anti-MCP-1/CCL2 antibody compared with CCR2$^{-/-}$ mice; pulmonary and lymph-node immune responses assessed	MCP-1/CCL2 neutralization and CCR2 genetic deletion	MCP-1/CCL2 neutralization and CCR2 deficiency produce overlapping but nonidentical immune defects	MCP-1/CCL2 primarily supports leukocyte recruitment into infected lungs, whereas CCR2 has broader roles in antigen-presenting cell trafficking and Th1 immune development	Strong [5]
C. neoformans	Lung	CCR2$^{+/+}$ and CCR2$^{-/-}$ mice infected intratracheally; pulmonary CCL2, CCL7, and CCL12 mRNA measured by RT-PCR**; dendritic-cell recruitment and bronchovascular infiltrates assessed	CCR2 genetic deletion	CCL2, CCL7, and CCL12 are induced together during pulmonary infection	MCP-1/CCL2 functions within a broader CCR2-ligand network that supports dendritic-cell recruitment and formation of bronchovascular mononuclear infiltrates	Moderate [6]
C. neoformans	Lung	WT, B-cell-deficient, FcRγIII-deficient, athymic, and SCID# mice; lung MCP-1/CCL2 protein measured by ELISA	Anti-GXM## antibody treatment across different immune backgrounds	MCP-1/CCL2 increases during infection and is particularly elevated in some B-cell- and Fc receptor-deficient settings; antibody-associated changes vary with host background	Elevated MCP-1/CCL2 does not consistently indicate protective immunity and may accompany dysregulated inflammation depending on host immune status	Weak [7]
C. neoformans	Lung	BALB/c mice infected intratracheally; lung MCP-1/CCL2 protein measured by ELISA after neutrophil depletion	Anti-neutrophil antibody	Infection increases pulmonary MCP-1/CCL2 protein, but levels remain similar between neutrophil-depleted and control mice	Neutrophil-dependent changes in disease outcome occur without measurable changes in MCP-1/CCL2, indicating that MCP-1/CCL2 is not the major mediator of this phenotype	Weak [8]
C. neoformans	Lung	Mice infected intratracheally; lung MCP-1/CCL2 mRNA measured by RT-PCR and lung homogenate protein by ELISA	IL-12 treatment with or without IFN-γ neutralization	IL-12 markedly increases MCP-1/CCL2 mRNA and protein, whereas IFN-γ neutralization reduces this induction	MCP-1/CCL2 lies downstream of an IL-12/IFN-γ-associated protective immune pathway that supports mononuclear leukocyte recruitment	Moderate [9]
C. neoformans	Lung	Mice infected intratracheally with a highly virulent strain; pulmonary MCP-1/CCL2 mRNA measured by RT-PCR and BALF protein by ELISA	IL-12 treatment	IL-12 induces MCP-1/CCL2 mRNA by day 3 and increases BALF MCP-1/CCL2 protein at days 7 and 14	Increased MCP-1/CCL2 accompanies macrophage and CD4^{+} T-cell accumulation, supporting its participation in IL-12-associated protective immunity	Moderate [10]

C. neoformans	Lung	CBA/J mice infected intratracheally; BALF MCP-1/CCL2 protein measured by ELISA after transient TNF-α blockade	TNF-α neutralization during early infection	Early TNF-α blockade reduces MCP-1/CCL2 at day 7, but MCP-1/CCL2 recovers by day 14	Early disruption of MCP-1/CCL2-associated immune programming is followed by persistent infection despite later chemokine recovery, emphasizing the importance of timing	Moderate [11]
C. neoformans	Lung / granulomas	Rats followed during persistent pulmonary cryptococcosis; lung MCP-1/CCL2 protein measured by ELISA and immunohistochemistry; pulmonary macrophages studied ex vivo	Opsonization and macrophage phagocytosis experiments ex vivo	Lung MCP-1/CCL2 protein peaks during early/subacute infection and declines during chronic disease; pulmonary macrophages and epithelioid cells are major local sources	MCP-1/CCL2 production is dynamically regulated during persistent infection and is induced directly by macrophage interaction with opsonized *cryptococci*	Moderate [12]
Cryptococcus deneoformans	Lung	C57BL/6 and 5-LO-deficient mice infected intranasally; lung cytokine/chemokine protein measured by Bio-Plex	5-LO genetic deletion	Lung MCP-1/CCL2 protein is increased in 5-LO-deficient mice at day 7, with no difference by day 14	MCP-1/CCL2 regulation varies with infection stage and tissue context rather than following a uniform inflammatory pattern	Moderate [13]
C. neoformans	Pulmonary-relevant human leukocytes	Human blood monocytes and bronchoalveolar macrophages stimulated with *C. neoformans*; MCP-1/CCL2 mRNA measured by Northern blot and secreted protein by ELISA	Serum/complement manipulation during fungal stimulation	Human blood monocytes strongly induce MCP-1/CCL2 mRNA and protein, whereas bronchoalveolar macrophages show little MCP-1/CCL2 induction	Recruited monocytes may contribute more strongly than resident bronchoalveolar macrophages to MCP-1/CCL2 amplification after pulmonary cryptococcal exposure	Moderate [14]
C. neoformans	Pulmonary-relevant human mast cells	Human HMC-1 mast cells exposed to *C. neoformans*; MCP-1/CCL2 protein measured by multiplex assay and ELISA; monocyte migration assessed by Transwell assay	Syk inhibition	*C. neoformans* induces dose-dependent MCP-1/CCL2 release, while Syk inhibition reduces MCP-1/CCL2 production	Mast-cell-derived MCP-1/CCL2 may contribute to early monocyte recruitment following cryptococcal exposure	Moderate [15]
C. neoformans	Human bronchial epithelium	BEAS-2B cells exposed to *C. neoformans*, *Dermatophagoides pteronyssinus*, or both; secreted MCP-1/CCL2 protein measured by ELISA	Fungal and allergen co-exposure	*C. neoformans* alone does not significantly increase MCP-1/CCL2 protein and suppresses allergen-induced MCP-1/CCL2 production	*C. neoformans* suppresses epithelial MCP-1/CCL2-associated inflammatory signaling under selected co-exposure conditions	Weak [16]

Cryptococcus gattii	Human bronchial epithelium	BEAS-2B cells exposed to live or heat-killed *C. gattii* with IL-4, IFN-γ, or IL-27; CCL2 protein measured by ELISA	Cytokine pretreatment and altered order of fungal exposure	Live *C. gattii* alone does not strongly induce MCP-1/CCL2 but suppresses IL-4- and IL-27-induced MCP-1/CCL2 when cytokine exposure precedes infection	Epithelial MCP-1/CCL2 suppression depends on fungal viability and timing of exposure, suggesting context-dependent modulation of airway chemokine responses	Weak [17]
C. neoformans	Lung	BALB/c mice infected with melanized or nonmelanized organisms, melanin ghosts, or laccase-deficient/complemented strains; lung MCP-1/CCL2 protein measured by ELISA	Fungal melanization and laccase manipulation	Melanized organisms and melanin ghosts increase MCP-1/CCL2; laccase-positive organisms induce more MCP-1/CCL2 than laccase-deficient organisms	Cryptococcal melanin and laccase activity directly shape the magnitude of pulmonary MCP-1/CCL2-associated inflammation	Moderate [18]
C. neoformans	Lung	BALB/c mice infected intranasally with virulent H99 or a calcineurin A mutant at different inocula; lung MCP-1/CCL2 protein measured by ELISA	Fungal strain and inoculum manipulation	MCP-1/CCL2 production varies with fungal strain and inoculum size; high-dose mutant and H99 infection induce greater MCP-1/CCL2	Increased MCP-1/CCL2 accompanies cellular recruitment without being sufficient to produce effective protective immunity	Moderate [19]
C. neoformans	Lung	MARCO$^{+/+}$ and MARCO$^{-/-}$ mice infected with *C. neoformans*; pulmonary MCP-1/CCL2 mRNA measured by RT-qPCR and MCP-1/CCL2 protein by ELISA	MARCO genetic deletion	MARCO deficiency reduces early pulmonary MCP-1/CCL2 mRNA and protein	MARCO contributes to early MCP-1/CCL2 induction and the inflammatory recruitment associated with fungal containment	Moderate [20]
C. neoformans	Lung	C57BL/6 mice infected with H99; pulmonary MCP-1/CCL2 mRNA measured by real-time PCR	Broad-spectrum MMP inhibitor GM6001	MMP inhibition reduces pulmonary MCP-1/CCL2 mRNA, while MMP-12 expression correlates with MCP-1/CCL2 expression	MMP-associated inflammation contributes to MCP-1/CCL2 induction and inflammatory-cell recruitment, although MCP-1/CCL2 itself is not directly manipulated	Moderate [21]
C. neoformans	Lung and primary alveolar macrophages	Mice infected with virulent H99 or granuloma-forming Δgcs1 strain; WT and SK1-deficient mice compared; BALF and macrophage MCP-1/CCL2 protein measured	Fungal Δgcs1 mutation; host SK1 deletion	Δgcs1 infection induces higher BALF MCP-1/CCL2 than H99, while SK1 deficiency reduces MCP-1/CCL2 in vivo and in primary macrophages	SK1/S1P signaling promotes MCP-1/CCL2 production during granuloma-associated fungal containment	Moderate [22]

C. neoformans	Lung	WT and IL-4 receptor alpha-deficient mice infected intranasally; pulmonary CCL2 mRNA measured by RT-qPCR	IL-4 receptor alpha genetic deletion	IL-4 receptor alpha deficiency reduces pulmonary CCL2 mRNA, particularly at day 14	IL-4 receptor alpha signaling contributes to early MCP-1/CCL2 expression and recruitment of alveolar macrophages and dendritic cells	Moderate [23]
C. neoformans	Murine macrophages	Murine peritoneal macrophages stimulated with CpG[$] ODN[$$] with or without IFN-γ priming; MCP-1/CCL2 protein measured after stimulation	TLR9 stimulation with CpG ODN	CpG stimulation increases macrophage MCP-1/CCL2 protein production in vitro	TLR9 activation induces MCP-1/CCL2 as part of a broader innate activation program, but its contribution to cryptococcal protection is not directly tested	Weak [24]
C. neoformans	Human monocytes/macrophages	Human THP-1 monocytes treated with *Pleurotus pulmonarius* crude extract differentiated macrophages subsequently challenged with opsonized *C. neoformans*; mRNA measured by RT-qPCR	*Pleurotus pulmonarius* crude-extract pretreatment	Crude-extract treatment increases MCP-1/CCL2 mRNA in THP-1 monocytes and in extract-trained macrophages subsequently challenged with *C. neoformans*	Increased MCP-1/CCL2 transcription accompanies immunomodulatory conditioning and a shift away from an M2-associated phenotype, without establishing that MCP-1/CCL2 mediates the polarization change	Weak [25]

*ELISA = Enzyme-linked immunosorbent assay

**(q)RT-PCR = (Quantitative) reverse transcription real-time polymerase chain reaction

#SCID = Severe combined immunodeficiency

##GXM = Glucuronoxylomannan

$CpG = Cytosine-phosphate-guanosine

$$ODN = Oligodeoxynucleotides

Table S2. MCP-1/CCL2 evidence in CNS cryptococcosis and human cryptococcal meningitis

Fungal species	Infection site	Experimental model	Functional intervention	MCP-1/CCL2 response	Interpretation	Strength of evidence
C. neoformans	Brain	C57BL/6J mice infected by retro-orbital intravenous inoculation; brain MCP-1/CCL2 protein measured by CBA*; inflammatory monocytes and neuropathology assessed	CCR2 genetic deletion	Brain MCP-1/CCL2 protein increases beginning around day 14 and peaks around day 21 during the inflammatory phase of infection	CCR2-dependent inflammatory monocyte recruitment contributes to brain infiltration and neuropathology, although the CCR2 phenotype is not specific to MCP-1/CCL2 alone	Moderate [26]
C. neoformans	Brain and microglia	Restraint-stress mouse model; isolated microglia cultured ex vivo; MCP-1/CCL2 protein measured by ELISA; brain fungal burden assessed after recombinant MCP-1/CCL2 or anti-MCP-1/CCL2 treatment	Recombinant MCP-1/CCL2 administration; direct anti-MCP-1/CCL2 neutralization	Microglia from restraint-stressed mice produce MCP-1/CCL2, whereas control microglia show no detectable production	Recombinant MCP-1/CCL2 increases brain fungal burden, while MCP-1/CCL2 neutralization reduces fungal burden in stressed mice, directly supporting a pathologic role under stress-associated immune dysregulation	Strong [27]
C. deneoformans	Brain	C57BL/6 and 5-LO-deficient mice infected intranasally; brain cytokine and chemokine protein measured by Bio-Plex; leukocyte infiltration and fungal burden assessed	5-LO genetic deletion	Brain MCP-1/CCL2 protein is reduced in 5-LO-deficient mice during late infection and accompanies reduced leukocyte infiltration	5-LO signaling contributes to sustained MCP-1/CCL2-associated CNS inflammation, while its effect differs between early lung and late brain infection	Moderate [13]
C. neoformans	Brain	WT and IL-23p19-deficient mice infected with *C. neoformans*; brain MCP-1/CCL2 mRNA measured by qRT-PCR; protein measured by Luminex	IL-23 p19 genetic deletion	IL-23 p19 deficiency reduces both brain MCP-1/CCL2 mRNA and protein during infection	IL-23 signaling promotes CNS MCP-1/CCL2 production as part of the inflammatory response to cryptococcal infection	Moderate [28]
C. neoformans	Brain and meninges	Fischer rats infected intracisternally with smooth or mucoid variants; brain protein measured by ELISA; intracranial pressure,	Fungal phenotypic switching	The mucoid variant induces substantially higher brain MCP-1/CCL2 protein than the smooth parent strain	Increased MCP-1/CCL2 accompanies greater monocyte/macrophage infiltration, cryptococcal polysaccharide	Moderate [29]

		inflammation, and survival assessed			accumulation, elevated intracranial pressure, and shorter survival	
C. neoformans	Brain	Mice immunized with cryptococcal culture filtrate antigen and challenged intracerebrally; CNS MCP-1/CCL2 mRNA and protein assessed with broader cytokine and chemokine responses	Protective immunization before intracerebral challenge	MCP-1/CCL2 increases together with a broader Th1-associated cytokine and chemokine response in immunized mice	MCP-1/CCL2 participates in protective CNS inflammation when recruitment occurs within a coordinated cell-mediated antifungal response	Weak [30]
C. neoformans	Brain, lung, spleen, and plasma	TNF/LT-α-deficient and WT mice infected intravenously; MCP-1/CCL2 protein measured by ELISA across tissue compartments	TNF/LT-α genetic deficiency	MCP-1/CCL2 is higher in plasma and lung, lower in spleen, and similar in brain in deficient mice compared with WT mice	MCP-1/CCL2 regulation is compartment-specific, and increased levels in selected tissues do not compensate for loss of broader TNF/LT-α-dependent antifungal immunity	Moderate [31]
Cryptococcus spp.	Cerebrospinal fluid	Prospective cohort of patients with HIV-associated cryptococcal meningitis; baseline CSF cytokines measured before subsequent clinical follow-up	None	Baseline CSF MCP-1/CCL2 is higher in patients who subsequently develop C-IRIS	Elevated baseline CSF MCP-1/CCL2 is associated with later IRIS and supports its use as part of an inflammatory risk profile	Weak [32]
Cryptococcus spp.	Cerebrospinal fluid and plasma	Prospective cohort of patients with HIV-associated cryptococcal meningitis treated with antifungal therapy followed by cART; CSF and plasma chemokines assessed	None	CSF MCP-1/CCL2 is higher than plasma MCP-1/CCL2, and a higher CSF MCP-1/CCL2 to CXCL10 ratio is associated with later C-IRIS	CNS MCP-1/CCL2 enrichment reflects compartmentalized inflammation, while the MCP-1/CCL2 to CXCL10 balance may identify a myeloid-biased inflammatory state associated with IRIS	Weak [33]
Cryptococcus spp.	Cerebrospinal fluid	Non-HIV cryptococcal meningitis cohort; baseline CSF cytokines and LTA4H polymorphisms assessed	None	Baseline CSF MCP-1/CCL2 is higher in patients who subsequently develop IRIS and is associated with LTA4H polymorphisms	MCP-1/CCL2-associated IRIS risk extends beyond HIV-associated disease and may interact with leukotriene-related inflammatory regulation	Weak [34]
Cryptococcus spp.	Cerebrospinal fluid	HIV-associated cryptococcal meningitis cohort stratified by baseline $CD4^+$ T-cell count; CSF cytokines and chemokines measured	None	Patients with $CD4^+$ T-cell counts of at least 100 cells/μL have lower CSF MCP-1/CCL2 despite higher concentrations of several other inflammatory mediators	CSF MCP-1/CCL2 varies with host immune status and does not increase uniformly with stronger CNS inflammation	Weak [35]

Cryptococcus spp.	Cerebrospinal fluid	HIV-associated cryptococcal meningitis cohort stratified by altered mental status at presentation; CSF inflammatory markers and opening pressure assessed	None	Patients with altered mental status have lower CSF MCP-1/CCL2 than patients with normal mental status	Lower CSF MCP-1/CCL2 may accompany severe neurologic disease and supports interpretation of the chemokine as part of an immune phenotype rather than a simple severity marker	Weak [36]
Cryptococcus spp.	Cerebrospinal fluid	Patients with HIV and cryptococcal meningitis, tuberculous meningitis, or no meningitis; CSF cytokine and chemokine profiles compared	None	CSF MCP-1/CCL2 is higher in cryptococcal meningitis than in tuberculous meningitis	MCP-1/CCL2 contributes to disease-associated differences in CNS inflammatory profiles but does not establish an MCP-1/CCL2-specific mechanism	Weak [37]
Cryptococcus spp.	Cerebrospinal fluid	Patients with HIV with cryptococcal meningitis, toxoplasmic encephalitis, or no CNS opportunistic infection; CSF chemokines measured by ELISA	None	CSF MCP-1/CCL2 is higher in cryptococcal meningitis than in HIV without CNS opportunistic infection but is not significantly higher than in toxoplasmic encephalitis	MCP-1/CCL2 reflects broader CNS opportunistic infection-associated inflammation rather than a cryptococcus-specific inflammatory signature	Weak [38]
Cryptococcus spp.	Cerebrospinal fluid	Patients with AIDS-associated cryptococcal meningitis; CSF cytokines measured at baseline and during treatment	None	CSF MCP-1/CCL2 is elevated in cryptococcal meningitis compared with healthy controls and occurs within a broader inflammatory cytokine response	MCP-1/CCL2 reflects CNS inflammation but does not emerge as a dominant stand-alone outcome marker	Weak [39]
Cryptococcus spp.	Cerebrospinal fluid	Longitudinal cytokine analysis from the CryptoDex randomized trial in HIV-associated cryptococcal meningitis	None	Baseline CSF MCP-1/CCL2 is associated with 10-week clinical outcome but not 6-month outcome, while dexamethasone-associated harm is more closely related to accelerated TNF-α decline	MCP-1/CCL2 contributes to the clinical inflammatory profile but does not appear to be the main pathway explaining dexamethasone-associated harm	Weak [40]
Cryptococcus spp.	Plasma, with disseminated lung and CNS disease	Patients with HIV-associated disseminated cryptococcosis involving the lungs and CNS; baseline plasma cytokines measured before treatment and survival followed for 3 years	None	Survivors have lower baseline plasma MCP-1/CCL2 than patients who die during follow-up	Elevated circulating MCP-1/CCL2 is associated with worse long-term prognosis and reflects systemic inflammatory burden rather than a CNS-specific mechanism	Weak [41]

*CBA = Cytometric bead array

Table S3. MCP-1/CCL2 evidence in *Candida*

Fungal species	Infection site	Experimental model	Functional intervention	MCP-1/CCL2 response	Interpretation	Strength of evidence
Candida albicans	Vagina	Estrogen-treated CBA/J mice infected intravaginally; vaginal tissue and draining lymph nodes analyzed by RT-PCR and ELISA	Direct anti-MCP-1/CCL2 neutralization	Vaginal MCP-1/CCL2 protein rises by day 2 and mRNA from day 4, while draining lymph-node protein does not increase	Neutralization increases vaginal fungal burden, directly supporting a protective local MCP-1/CCL2 response	Strong [42]
C. albicans	Oral and vaginal epithelium	Primary human epithelial cells and epithelial cell lines exposed to *C. albicans*; supernatant protein measured by ELISA	Fungal exposure only	Oral and vaginal epithelial cells secrete MCP-1/CCL2 protein after fungal exposure	Mucosal epithelial cells are local sources of MCP-1/CCL2	Weak [43]
C. albicans	Gingival epithelium	Rat gingival infection analyzed by microarray; RT-PCR, and immunohistochemistry	Experimental gingival infection	MCP-1/CCL2 mRNA increases and protein localizes near invaded epithelium	Gingival epithelium contributes to local MCP-1/CCL2 production during fungal invasion	Weak [44]
C. albicans	Stomach	Amoxicillin-associated murine gastric infection; limiting ridge analyzed by qPCR, histology, and flow cytometry	Amoxicillin pretreatment before gastric infection	Local MCP-1/CCL2 mRNA increases together with monocyte/macrophage infiltration	MCP-1/CCL2 accompanies localized mononuclear-cell recruitment at sites of gastric invasion	Weak [45]
Candida glabrata	Osteoblasts	Human osteoblasts exposed to viable, heat-killed, opsonized, or nonopsonized yeast; supernatant protein measured by ELISA	Opsonization and fungal viability conditions	Opsonized viable *C. glabrata* induces the strongest MCP-1/CCL2 protein response	Osteoblasts contribute to local MCP-1/CCL2 production after fungal exposure	Weak [46]
C. albicans	Dental pulp stem cells	Human dental pulp stem cells stimulated with *C. albicans* and analyzed by single-cell RNA-seq*	Fungal stimulation only	A *Candida*-responsive subpopulation shows increased MCP-1/CCL2 transcription	Stromal progenitor cells participate in local MCP-1/CCL2 transcriptional responses	Weak [47]
C. albicans	Adipocytes	3T3-L1 adipocytes exposed to heat-killed fungi, β-glucan particles, or macrophage-conditioned medium; mRNA measured by qPCR	IKKβ inhibition during fungal-particle stimulation	Fungal-associated stimuli increase MCP-1/CCL2 mRNA, while IKKβ inhibition reduces induction	MCP-1/CCL2 induction depends partly on IKKβ-associated NF-κB signaling	Moderate [48]

C. albicans	Oral mucosa	WT and p40$^{-/-}$ mice with OPC; oral mRNA measured by qRT-PCR	IL-12/IL-23 shared p40 genetic deletion	WT mice show transient MCP-1/CCL2 mRNA induction, while p40$^{-/-}$ mice fail to induce it	Oral MCP-1/CCL2 induction depends on a p40-associated inflammatory pathway	Moderate [49]
C. albicans	Kidney and spleen	BALB/c mice infected with WT, mannosylation-deficient, and reintegrant strains; tissue protein measured by multiplex assay	Fungal mannosylation mutations	MCP-1/CCL2 protein responses vary by mannosylation status and organ	Fungal cell-wall architecture shapes tissue-specific MCP-1/CCL2 induction	Moderate [50]
C. albicans	Endothelial cells	HMEC-1 cells infected with invasive WT, rim101 mutant, or complemented strain; cytokines measured in lysates and supernatants	Rim101 disruption and complementation	Invasive strains induce MCP-1/CCL2, while the invasion-deficient mutant shows reduced induction	Fungal invasive capacity influences endothelial MCP-1/CCL2 responses	Moderate [51]
C. albicans	Kidney	Mice infected intravenously with WT, ECE1-deficient, or candidalysin-deficient strains; renal chemokines measured in homogenates	ECE1 deletion and candidalysin-specific disruption	Loss of ECE1 or candidalysin reduces early renal MCP-1/CCL2 protein	Candidalysin contributes to early renal MCP-1/CCL2 induction	Moderate [52]
C. albicans-derived CAWS	Heart and coronary arteries	CAWS arteritis model using MCP-1/CCL2 reporter mice; cardiac MCP-1/CCL2 transcription and cellular source assessed; pathway-deficient mice, monocyte transfer, and flow cytometry used to examine recruitment	Dectin-2 pathway disruption, CCR2-dependent monocyte transfer, and IL-1 pathway manipulation	CAWS induces early MCP-1/CCL2 transcription in cardiac tissue-resident macrophages followed by a later endothelial CCL2 response	Fungal mannan sensing establishes an MCP-1/CCL2-associated inflammatory recruitment circuit	Moderate [53]
C. albicans	Macrophages	BMDMs** infected with *C. albicans*; inflammatory genes measured by qPCR	Tim-3 or Galectin-9 antibody blockade	Blocking Tim-3 or Galectin-9 increases MCP-1/CCL2 mRNA	Galectin-9/Tim-3 signaling suppresses macrophage MCP-1/CCL2 responses	Moderate [54]
Candida spp.	Peripheral T cells	T cells from one STAT1 gain-of-function patient and one control stimulated with IFNα and IFNγ; mRNA measured by real-time PCR	IFNα and IFNγ stimulation	Patient T cells show greater IFN-induced MCP-1/CCL2 transcription	STAT1 gain-of-function may alter MCP-1/CCL2 regulation, but evidence is limited by the single-patient design	Weak [55]
C. albicans	Kidney and macrophages	WT and Trim72$^{-/-}$ mice with systemic candidiasis; kidney MCP-1/CCL2 protein measured by ELISA; renal leukocytes assessed	Trim72 genetic deletion; recombinant Trim72 treatment; macrophage	Trim72 increases MCP-1/CCL2 protein and macrophage recruitment,	MCP-1/CCL2 is functionally required for Trim72-mediated macrophage recruitment and protection	Strong [56]

		by flow cytometry; murine and human macrophage migration assays performed	depletion; Bindarit-mediated MCP-1/CCL2 inhibition; NF-κB or ERK1/2 inhibition	while MCP-1/CCL2 inhibition reverses protection		
C. albicans	Kidney and bone marrow-derived cells	WT and Ifnar1$^{-/-}$ mice with systemic candidiasis; renal mRNA and protein production assessed in bone marrow-derived cells; inflammatory monocyte recruitment analyzed	Ifnar1 genetic deletion; pioglitazone treatment	Ifnar1 deficiency reduces renal MCP-1/CCL2 mRNA, cellular MCP-1/CCL2 protein, and inflammatory monocyte recruitment	Type I IFN signaling promotes MCP-1/CCL2 production and monocyte trafficking	Moderate [57]
C. albicans	Human monocytes	Integrated GWAS[#], bulk RNA-seq, single-cell RNA-seq analyses followed by THP-1 LY86 knockdown; mRNA measured by qRT-PCR; migration toward MCP-1/CCL2 assessed by Transwell assay	LY86 siRNA knockdown	LY86 knockdown reduces CCR2 expression and migration toward MCP-1/CCL2	Candidemia susceptibility biology is linked to CCR2-dependent responsiveness to MCP-1/CCL2	Moderate [58]
C. albicans	Peritoneal macrophages during disseminated infection	Alloxan-induced diabetic mouse model with disseminated candidiasis; MCP-1/CCL2 protein in peritoneal macrophage supernatants measured by CBA	Alloxan-induced hyperglycemia	Hyperglycemia reduces macrophage MCP-1/CCL2 protein, especially during later infection	Impaired MCP-1/CCL2 production accompanies defective macrophage function and increased fungal burden	Moderate [59]
Candida spp.	Peripheral blood monocytes	Monocytes from patients with denture stomatitis and controls; supernatant protein measured by ELISA	LPS[##] stimulation ex vivo	Patient monocytes produce little detectable MCP-1/CCL2 under basal or stimulated conditions	Impaired MCP-1/CCL2 production accompanies chronic mucosal *Candida*-associated disease	Weak [60]
Candida dubliniensis	Bone marrow	*C. dubliniensis* immunization and trained-immunity model; bone-marrow extracellular-fluid MCP-1/CCL2 measured by Luminex	*C. dubliniensis* immunization	Bone marrow MCP-1/CCL2 protein increases during early myelopoiesis and trained immunity	MCP-1/CCL2 accompanies broader systemic myeloid programming, but its specific function is not tested	Weak [61]
C. albicans	Serum and kidney	Susceptible SM/J, resistant C57BL/6J, and F2 mice; serum protein measured by ELISA and renal	Comparison of resistant and susceptible strains with	Susceptible mice have higher serum MCP-1/CCL2 but fewer renal	High circulating MCP-1/CCL2 is insufficient for protection when	Weak [62]

		monocytes assessed by flow cytometry	Carg5 segregation analysis	inflammatory monocytes and greater fungal burden	downstream recruitment is ineffective	
C. albicans	Kidney	BALB/c mice infected intravenously with multiple strains; kidney protein measured by CBA	Comparison of multiple *C. albicans* strains with different virulence	Renal MCP-1/CCL2 protein increases early and is highest with more virulent strains	Elevated MCP-1/CCL2 accompanies greater fungal burden, tissue lesions, and leukocyte infiltration	Weak [63]
C. albicans	Serum	A/J, C57BL/6J, and congenic BcA17 mice with systemic candidiasis; serum cytokines measured by RayBio array	C5 deficiency and congenic C5 allele comparison	C5-deficient susceptible mice show increased circulating MCP-1/CCL2 protein signal after infection	C5 deficiency links elevated MCP-1/CCL2 with dysregulated inflammation and susceptibility	Moderate [64]
C. albicans	Kidney	Myeloid-specific PTP1B-deficient and control mice with systemic candidiasis; renal mRNA measured by RT-qPCR and leukocyte infiltration assessed by flow cytometry	Myeloid-specific PTP1B genetic deletion	PTP1B deficiency increases renal MCP-1/CCL2 mRNA and leukocyte infiltration	Increased MCP-1/CCL2 accompanies greater fungal burden and renal inflammation	Moderate [65]
C. albicans	Kidney	Macrophage-specific IL-1Ra-deficient and control mice with *Candida* sepsis; renal inflammatory gene expression assessed by bulk RNA-seq	Macrophage-specific IL-1Ra genetic deletion	Improved clearance is accompanied by lower renal MCP-1/CCL2 transcript abundance	Reduced MCP-1/CCL2 accompanies improved fungal clearance and resolution of inflammation	Moderate [66]
C. albicans	Kidney	CD169-DTR[$] mice depleted of renal resident macrophages before systemic infection; renal mRNA measured by qRT-PCR and leukocyte recruitment assessed by flow cytometry	Diphtheria toxin-mediated CD169-positive resident macrophage depletion	Depletion does not significantly reduce renal MCP-1/CCL2 mRNA or early monocyte recruitment	Preserved MCP-1/CCL2 and recruitment are insufficient without protective resident macrophage function	Moderate [67]
C. albicans	Cornea	BALB/c and athymic nude mice with experimental keratitis; corneal mRNA measured by qRT-PCR in vivo and in ex vivo corneal explants	Comparison of BALB/c and athymic nude mice	MCP-1/CCL2 mRNA rises rapidly in BALB/c corneas, while athymic mice show reduced induction and leukocyte recruitment	MCP-1/CCL2-associated recruitment aligns with inflammatory disease development in the cornea	Weak [68]

C. albicans	Macrophages and kidney	BMDMs stimulated with heat-killed *C. albicans*; mice with experimental candidemia	IDR-1018 treatment	IDR-1018 increases macrophage MCP-1/CCL2 protein while improving survival in vivo	MCP-1/CCL2 increases as part of a broader treatment-associated protective response	Weak [69]
C. albicans	Macrophages and intra-abdominal infection	RAW264.7 macrophages and mice with intra-abdominal candidiasis; mRNA and protein measured	SNH prophylaxis with TLR2 inhibition or knockdown	SNH increases MCP-1/CCL2 mRNA and protein through TLR2, p38, and NF-κB-associated signaling	MCP-1/CCL2 induction occurs within a broader macrophage-activating response associated with improved fungal control	Moderate [70]
C. albicans	Oral mucosa	Murine oral candidiasis using fluconazole-susceptible and resistant strains	DNase I plus aPDT	Treatment increases local MCP-1/CCL2 protein early, while later responses differ by strain	Treatment-associated MCP-1/CCL2 induction depends on fungal strain and treatment stage	Weak [71]
C. albicans	Vagina	Estrogen-dependent murine vaginal candidiasis; vaginal mRNA measured by qRT-PCR	BDSF treatment	BDSF reduces MCP-1/CCL2 mRNA together with fungal burden and inflammation	Declining MCP-1/CCL2 likely reflects resolution of fungal-driven mucosal inflammation	Weak [72]
C. albicans	Kidney	Murine disseminated candidiasis-associated AKI; renal mRNA/protein measured by RT-qPCR Western blot; miR-124 targeting assessed by luciferase reporter assay	Itraconazole treatment; miR-124 overexpression	Both interventions reduce renal MCP-1/CCL2 mRNA and protein	Reduced MCP-1/CCL2 accompanies improved renal function, tissue injury, and survival, with MCP-1/CCL2 identified as a miR-124 target	Moderate [73]
C. albicans	Vagina	Experimental vaginal candidiasis with adenoviral cytokine delivery	Adenoviral MCP-1/CCL2 delivery	Adenoviral delivery produces transient local MCP-1/CCL2 expression	Local delivery is feasible, but its effect on fungal burden is not directly tested	Weak [74]
C. albicans	Macrophages, indirect inflammatory model	LPS-stimulated macrophages evaluated during hinokitiol treatment	Hinokitiol treatment during LPS stimulation	Hinokitiol reduces LPS-induced MCP-1/CCL2 mRNA	MCP-1/CCL2 is pharmacologically modulated, but the effect is not tested during direct *Candida* stimulation	Weak [75]

*seq = Sequencing

**BMDMs = Bone marrow-derived macrophages

#GWAS = Genome-wide association study

##LPS = Lipopolysaccharide

[$]DTR = diphtheria toxin receptor

Table S4. MCP-1/CCL2 evidence in *Aspergillus*

Fungal species	Infection site	Experimental model	Functional intervention	MCP-1/CCL2 response	Interpretation	Strength of evidence
Aspergillus fumigatus	Cornea	Mouse fungal keratitis and macrophage functional assays; corneal mRNA/protein measured by RT-PCR/Western blot; macrophage polarization assessed by flow cytometry; phagocytosis and fungal killing assessed	IDO inhibition or activation; CCR2 antagonist, anti-MCP-1/CCL2 neutralization, recombinant MCP-1/CCL2	Infection increases MCP-1/CCL2 and CCR2. MCP-1/CCL2 neutralization or CCR2 blockade reduces macrophage phagocytosis and killing, while recombinant MCP-1/CCL2 enhances both	Direct evidence that MCP-1/CCL2-CCR2 signaling regulates macrophage antifungal function during keratitis	Strong [76]
A. fumigatus	Cornea	Rat fungal keratitis; corneal epithelium analyzed by real-time RT-PCR	Dectin-1 blockade with laminarin	Corneal MCP-1/CCL2 mRNA increases from 4 to 24 h, while laminarin reduces the early 4 h response	Early MCP-1/CCL2 induction is partly Dectin-1-dependent	Moderate [77]
A. fumigatus	Cornea	Rat fungal keratitis; corneal MCP-1/CCL2 mRNA measured by RT-PCR and protein localized by immunohistochemistry	Experimental corneal infection	MCP-1/CCL2 mRNA increases and protein localizes to corneal epithelium, but expression does not closely correlate with Mincle	MCP-1/CCL2 is part of the local corneal response but is not simply Mincle-dependent	Weak [78]
A. fumigatus	Cornea and macrophages	RAW264.7 macrophages and mouse keratitis model; mRNA/protein measured by qRT-PCR/ELISA; fungal burden and clinical severity assessed	Ebselen treatment	Ebselen reduces MCP-1/CCL2 mRNA and protein together with fungal burden, oxidative stress, and disease severity	MCP-1/CCL2 decreases as part of broader inflammatory resolution during treatment	Weak [79]
A. fumigatus	Cornea and corneal epithelial cells	Mouse keratitis and human corneal epithelial-cell model; mRNA/protein measured; fungal burden and inflammatory-cell infiltration assessed	Sanguinarine treatment	Sanguinarine reduces MCP-1/CCL2 mRNA and protein together with fungal burden and inflammatory-cell infiltration	Reduced MCP-1/CCL2 accompanies improvement in keratitis rather than establishing MCP-1/CCL2 as the therapeutic target	Weak [80]

A. fumigatus	Cornea	Murine fungal keratitis; corneal mRNA/protein measured; Dectin-1/LOX-1** expression and inflammatory-cell infiltration assessed	β-glucan-specific nanobody treatment	Nanobody treatment reduces Dectin-1, LOX-1, MCP-1/CCL2 mRNA/protein, and macrophage/neutrophil infiltration	Fungal cell-wall recognition contributes to MCP-1/CCL2-associated corneal inflammation	Weak [81]
A. fumigatus	Cornea and macrophages	Mouse keratitis and RAW264.7 macrophages; inflammatory mediators and Mincle/Syk signaling assessed; fungal burden and inflammatory outcomes measured	Pseudolaric acid B treatment	Treatment reduces MCP-1/CCL2 along with other inflammatory mediators and suppresses Mincle/Syk-associated signaling	MCP-1/CCL2 is part of the inflammatory program reduced during treatment	Weak [82]
A. fumigatus	Cornea and macrophages	Mouse keratitis and RAW264.7 macrophages; mRNA/protein measured; fungal burden, macrophage infiltration, and LOX-1 assessed	Perillaldehyde treatment	Treatment reduces MCP-1/CCL2 mRNA/protein together with fungal burden and LOX-1-associated inflammation	MCP-1/CCL2 decreases as part of broader treatment-associated inflammatory control	Weak [83]
A. fumigatus	Lung and serum	Murine aspergillosis vaccination model; serum/BAL cytokine profiling, survival, and fungal burden after challenge	Whole glucan particle vaccination	Vaccination increases MCP-1/CCL2 in serum and BALF before challenge and improves survival and fungal clearance	MCP-1/CCL2 is part of a broader vaccine-induced protective immune profile	Weak [84]
A. fumigatus	Lung and serum	Corticosteroid-immunosuppressed murine aspergillosis; survival, fungal burden, and serum cytokine profiling	Oral amphotericin B formulation with or without AHCC***	Treatment is associated with increased systemic MCP-1/CCL2 as part of broader immune activation	Beneficial therapy may increase MCP-1/CCL2 when immune activation accompanies fungal control	Weak [85]
A. fumigatus	Lung	Immunocompetent and neutropenic mice with intratracheal invasive pulmonary infection; lung ELISA, fungal burden, leukocyte flow cytometry, NK-cell transfer	Direct anti-MCP-1/CCL2 neutralization; NK-cell depletion; WT versus CCR2-deficient NK-cell transfer	Lung MCP-1/CCL2 protein rises early in neutropenic mice. Neutralization increases mortality and fungal burden and reduces NK-cell recruitment	Direct evidence that MCP-1/CCL2 protects through early CCR2-dependent NK-cell recruitment	Strong [86]
Aspergillus spp.	Lung	Neutropenic mice challenged with killed hyphae; lung	TNF neutralization	MCP-1/CCL2 protein rises rapidly after hyphal challenge and	MCP-1/CCL2 participates in a TNF-dependent amplification response associated with	Moderate [87]

		chemokines measured by ELISA/multiplex assay		is reduced by TNF neutralization	inflammatory dendritic-cell recruitment	
A. fumigatus	Lung	Neutropenic mice with invasive pulmonary infection; whole-lung MCP-1/CCL2 measured by ELISA	CCL17 or CCL22 neutralization; CCR4 genetic deletion	CCL17 neutralization and CCR4 deficiency increase pulmonary MCP-1/CCL2 and coincide with improved fungal control and survival	CCL17-CCR4 signaling restrains a protective MCP-1/CCL2-associated response	Moderate [88]
A. fumigatus	Lung	Murine solid-organ transplant-like infection; lung homogenate protein measured by Luminex	Hydrocortisone or tacrolimus plus hydrocortisone immunosuppression	Immunosuppressed mice show reduced early MCP-1/CCL2 followed by increased levels at 48 h when fungal burden is high	Later MCP-1/CCL2 elevation is insufficient to compensate for impaired antifungal immunity and instead accompanies uncontrolled fungal growth	Weak [89]
A. fumigatus	Lung	Mice exposed to chlorine gas before intratracheal infection; lung protein measured by Bio-Plex	Chlorine gas exposure	Chlorine-exposed infected mice have higher lung MCP-1/CCL2 together with greater fungal burden and inflammation	Increased MCP-1/CCL2 does not restore protection when host defense is impaired and instead accompanies severe inflammatory disease	Weak [90]
A. fumigatus	Lung	Neutropenic rats with unilateral invasive pulmonary infection; lung and serum cytokines plus fungal-load markers assessed	Amphotericin B treatment	MCP-1/CCL2 rises locally in infected lung but not serum or contralateral lung and decreases with treatment	Persistent local MCP-1/CCL2 tracks fungal invasion and inflammatory burden rather than compensating for neutropenia	Weak [91]
A. fumigatus	Lung	Nonsensitized and sensitized mice challenged with live conidia; lung ELISA, airway physiology, histology, fungal retention	Early or late anti-MCP-1/CCL2 neutralization; adenoviral MCP-1/CCL2 overexpression	Early MCP-1/CCL2 promotes macrophage recruitment and fungal clearance, while late MCP-1/CCL2 contributes to allergic inflammation after fungal control	Direct evidence for a time-dependent shift from protective to pathologic MCP-1/CCL2 activity	Strong [92]
A. fumigatus	Lung	Sensitized CCR2$^{+/+}$ and CCR2$^{-/-}$ mice challenged with live conidia; lung protein, BAL, histology, fungal persistence	CCR2 genetic deletion	CCR2$^{-/-}$ mice have elevated pulmonary MCP-1/CCL2 but impaired fungal clearance and exaggerated allergic inflammation	MCP-1/CCL2 abundance is insufficient when CCR2-dependent recruitment is lost	Moderate [93]

Aspergillus antigen	Lung	WT, MCP-1/CCL2-deficient, and CCR2-deficient mice repeatedly exposed to nonviable antigen; BAL and airway pathology assessed	MCP-1/CCL2 or CCR2 genetic deletion	Robust type 2 inflammation, airway hyperreactivity, mucus production, and fibrosis develop despite loss of MCP-1/CCL2 or CCR2	MCP-1/CCL2-CCR2 signaling is dispensable in this antigen-only allergic airway model	Strong [94]
A. fumigatus	Lung	WT and STAT6-deficient mice with chronic fungal asthma; lung MCP-1/CCL2 protein measured during chronic disease	STAT6 genetic deletion	STAT6 deficiency markedly reduces MCP-1/CCL2, but airway hyperresponsiveness and fibrosis still develop later	STAT6 supports chronic MCP-1/CCL2 production, but MCP-1/CCL2 is not required for all remodeling features	Moderate [95]
Aspergillus spp.	Airways	Human SAFS patients, atopic asthma controls, and healthy controls; candidate SNP genotyping	Genetic association analysis	Two MCP-1/CCL2 polymorphisms are associated with SAFS	Provides indirect human genetic evidence linking MCP-1/CCL2 variation with fungal-associated allergic airway disease	Weak [96]
Aspergillus spp.	Sinonasal mucosa	Nasal biopsies from dogs with naturally occurring sinonasal aspergillosis; qRT-PCR	Naturally occurring infection	MCP-1/CCL2 mRNA and other MCP-family chemokines are elevated in infected nasal tissue	MCP-1/CCL2 accompanies chronic local inflammatory recruitment	Weak [97]
Fungal-associated rhinosinusitis	Sinonasal mucosa	Human AFRS[#], CRSwNP[##], and control sinus biopsies; immunohistochemistry and tissue ELISA	None	MCP-1/CCL2 protein, CCR2-positive cells, and dendritic-cell markers are elevated in AFRS and CRSwNP	Supports associative local MCP-1/CCL2-CCR2 recruitment in chronic sinonasal inflammation	Weak [98]
A. fumigatus	Whole blood	Human lepirudin-anticoagulated whole blood stimulated with conidia; multiplex cytokine assay	CD14-positive monocyte depletion and CD15-positive granulocyte depletion	Conidia induce MCP-1/CCL2 protein, but depletion of either cell population does not significantly reduce release	Early human MCP-1/CCL2 production is not attributable solely to monocytes or granulocytes	Weak [99]
A. fumigatus	Alveolar epithelial and endothelial compartments	Human invasive aspergillosis-on-chip with epithelial cells, endothelial cells, and macrophages; compartment-specific supernatants analyzed	Presence or absence of macrophages and fungal infection	MCP-1/CCL2 protein increases during infection, especially in the endothelial compartment and with macrophages present	Supports multicellular and compartment-specific MCP-1/CCL2 production at the pulmonary interface	Weak [100]

Aspergillus protease	Airway epithelial cells	Human A549 airway epithelial cells exposed to *Aspergillus* protease; mRNA/protein measured by RT-qPCR/CBA; mitochondrial ROS inhibited with Mito-TEMPO	Mito-TEMPO mitochondrial ROS scavenging	Protease increases MCP-1/CCL2 mRNA and protein, while Mito-TEMPO reduces both	Fungal protease induces epithelial MCP-1/CCL2 through mitochondrial ROS-dependent signaling	Moderate [101]
A. fumigatus	Human monocytes	Human monocytes stimulated with fungal ligands or heat-inactivated *A. fumigatus*; mRNA measured by qPCR; protein response assessed	Cyclosporine A-mediated calcineurin inhibition	Fungal stimulation induces MCP-1/CCL2, while cyclosporine A suppresses the response	Calcineurin-NFAT signaling supports monocyte MCP-1/CCL2 production during fungal stimulation	Moderate [102]
A. fumigatus and *C. albicans*	Neutrophils	Human primary neutrophils and HL-60-derived neutrophil-like cells stimulated with fungal morphotypes; RNA-seq/qPCR and ELISA; conditioned-medium monocyte migration assessed by Transwell assay	Cyclosporine A or FK506-mediated calcineurin inhibition	Fungal stimulation induces MCP-1/CCL2, while calcineurin inhibitors suppress induction and reduce monocyte chemoattraction	Calcineurin-NFAT signaling supports neutrophil chemokine production and downstream immune-cell recruitment	Moderate [103]
Aspergillus spp.	Serum	High-risk hematology and HSCT[$] patients; prospective baseline serum cytokine profiling by Luminex	None	Higher baseline serum MCP-1/CCL2 predicts later invasive fungal disease and remains an independent risk factor	MCP-1/CCL2 serves as a risk-associated biomarker rather than direct evidence of causality	Weak [104]
Aspergillus spp.	Pulmonary invasive aspergillosis	Hematologic patients; candidate-gene polymorphism and interaction analysis	None	CCL2 and CCR2 polymorphisms appear in interaction models with Dectin-1 and Dectin-2 variants	Suggests interaction between the MCP-1/CCL2-CCR2 axis and fungal-recognition pathways in susceptibility	Weak [105]
Aspergillus spp.	Hematologic patients	Pediatric cases and controls; targeted resequencing of innate-immunity genes	None	CCL2 rs4586 associates with aspergillosis, while CCR2 rs1799865 shows a weaker association	Supports possible genetic susceptibility involving the MCP-1/CCL2-CCR2 axis without functional validation	Weak [106]

*CFU = Colony-forming unit

**LOX-1 = Lectin-like oxidized low-density lipoprotein receptor-1

***AHCC = Active hexose correlated compound

#AFRS = Allergic fungal rhinosinusitis (AFRS)
##CRSwNP = Chronic rhinosinusitis with nasal polyps
$HSCT = Hematopoietic stem cell transplantation

Table S5. MCP-1/CCL2 evidence in *other fungal species*

Fungal species	Infection site	Experimental model	Functional intervention	MCP-1/CCL2 response	Interpretation	Strength of evidence
Pneumocystis carinii	Lung and alveolar epithelium	WT and CD4-depleted mice plus primary type II alveolar epithelial cells; BALF and epithelial protein measured by ELISA; epithelial mRNA measured by RPA*; tissue localization assessed by in situ hybridization	JNK and IKK inhibition; TNFR deficiency; purified β-glucan comparison	MCP-1/CCL2 protein rises rapidly in BALF and localizes mainly to alveolar epithelial cells. Fungal stimulation induces epithelial mRNA and protein through JNK and IKK signaling, while β-glucan alone is insufficient	Alveolar epithelial cells are an important early source of MCP-1/CCL2, with induction requiring defined intracellular signaling beyond β-glucan alone	Moderate [107]
Pneumocystis spp.	Lung and alveolar epithelium	Primary WT and signaling-deficient alveolar epithelial cells plus infected mice; mRNA/protein measured by RPA/ELISA	Genetic disruption of MyD88, TRIF**, TLR2, TLR4, or IL-1R	MCP-1/CCL2 mRNA and protein require IL-1R and MyD88 but not TLR2, TLR4, or TRIF. Early BALF MCP-1/CCL2 is reduced in MyD88-deficient mice	Defines IL-1R/MyD88 signaling as an upstream regulator of early epithelial MCP-1/CCL2 production	Moderate [108]
Pneumocystis spp.	Lung	WT and MyD88$^{-/-}$ mice during immune clearance and CD4-depleted pneumonia. BALF protein measured by ELISA; leukocyte recruitment assessed	MyD88 genetic deletion and CD4 depletion	MyD88 deficiency lowers MCP-1/CCL2 during active pneumonia but increases and prolongs MCP-1/CCL2 during immune-mediated clearance	MyD88 regulation of MCP-1/CCL2 changes with immune status and disease stage	Moderate [109]
P. carinii	Lung	TNFR1/TNFR2 bone marrow chimeric mice with CD4 depletion and pulmonary infection; BALF and lung protein/ mRNA measured by ELISA/qRT-PCR	Selective TNFR deficiency in hematopoietic or lung parenchymal compartments	TNFR signaling in lung parenchymal cells promotes high MCP-1/CCL2 mRNA and protein together with increased macrophage recruitment	Parenchymal TNFR signaling promotes MCP-1/CCL2-associated inflammation, but greater MCP-1/CCL2 does not improve fungal control	Moderate [110]
P. carinii	Lung	Naturally infected SCID mice with or without spleen-cell immune reconstitution; lung MCP-1/CCL2 mRNA	Spleen-cell immune reconstitution	MCP-1/CCL2 mRNA remains low despite increasing organism burden in unreconstituted mice but rises sharply after immune	MCP-1/CCL2 more closely reflects immune-mediated pulmonary inflammation than fungal burden alone	Weak [111]

		measured by multitemplate RPA		reconstitution and declines after clearance		
Pneumocystis spp.	Lung	Patients with Pneumocystis pneumonia associated with AIDS, autoimmune disease, or malignancy; BALF cytokines profiling performed	None	BALF MCP-1/CCL2 varies by underlying immune condition and correlates with CRP[#] and LDH[##] rather than fungal burden measures alone	Human MCP-1/CCL2 levels are strongly shaped by host immune status	Weak [112]
Pneumocystis jirovecii	Lung and blood	Non-AIDS immunocompromised patients with PJP; BALF and blood collected at diagnosis; inflammatory mediators measured by ELISA/immunoassay	None	BALF and blood MCP-1/CCL2 protein levels are elevated in PJP; the BALF MCP-1/CCL2 to TGF-β1 ratio is more closely associated with mechanical ventilation and mortality	MCP-1/CCL2 is more informative as part of the pulmonary inflammatory balance than as an isolated severity marker, while concurrent infection further limits disease-specific interpretation	Weak [113,114]
Histoplasma capsulatum	Lung and spleen	WT, MCP-1/CCL2-deficient, and CCR2-deficient mice infected intranasally; fungal burden and leukocyte recruitment assessed	MCP-1/CCL2 genetic deletion, CCR2 genetic deletion, and CCL7 or CCL12 neutralization	MCP-1/CCL2 loss causes recruitment defects and transiently impaired fungal control. Combined loss of MCP-1/CCL2 and CCL7 markedly worsens infection	MCP-1/CCL2 contributes directly to protection but functions redundantly with CCL7 through CCR2-dependent recruitment	Strong [115]
H. capsulatum	Lung	Mice pretreated with erythropoietin before infection; pulmonary MCP-1/CCL2 protein and disease outcome assessed	Erythropoietin pretreatment	Erythropoietin increases pulmonary MCP-1/CCL2 together with inflammation and mortality	Elevated MCP-1/CCL2 accompanies harmful inflammation but is not demonstrated to drive the adverse phenotype	Weak [116]
Blastomyces dermatitidis	Respiratory mucosa and draining lymphoid tissue	Murine respiratory vaccination model using attenuated *B. dermatitidis*; $CCR2^{+}$ $Ly6C^{hi}$ inflammatory-monocyte recruitment assessed by flow cytometry; chemotaxis and calcium flux measured; MMP2	MMP2 inhibition or deletion; recombinant CCL7 treatment; CCR2 deficiency; $Ly6C^{hi}$ inflammatory-monocyte transfer	Vaccination impairs $Ly6C^{hi}$ monocyte migration and calcium flux in response to CCL2 and is associated with inactive serum CCL7. MMP2 inhibition or deletion improves monocyte recruitment, while	*B. dermatitidis* disrupts the broader MCP-1/CCL2/CCL7-CCR2 recruitment pathway, trapping inflammatory monocytes in bone marrow and weakening pulmonary T-cell priming. The study supports CCR2-ligand	Moderate [117]

		activity assessed by zymography		recombinant CCL7 restores CCR2-positive cell egress	dysfunction but does not isolate an MCP-1/CCL2-specific causal effect	
Paracoccidioides brasiliensis	Lung	WT and IFN-$\gamma^{-/-}$ mice infected with yeast ; lung mRNA measured by RT-PCR; lung-homogenate protein measured by ELISA	IFN-γ genetic deletion	WT mice show earlier MCP-1/CCL2 mRNA induction and higher early protein together with greater mononuclear-cell recruitment	IFN-γ promotes early pulmonary MCP-1/CCL2 production associated with granulomatous cellular recruitment	Moderate [118]
P. brasiliensis	Lung	BALB/c mice infected intranasally with viable conidia; serial lung histopathology performed; protein measured by Bio-Plex/Luminex bead assay	Longitudinal infection time course	MCP-1/CCL2 protein is elevated from 2 h to 4 weeks and returns toward baseline during later chronic pathology	MCP-1/CCL2 is most prominent during early pulmonary inflammation rather than peak chronic disease	Weak [119]
P. brasiliensis	Macrophages	WT and IL-10$^{-/-}$ peritoneal macrophages infected viable yeast; supernatant protein measured by ELISA; phagocytosis, nitric oxide, and fungal killing assessed	IL-10 genetic deletion	IL-10-deficient macrophages produce more MCP-1/CCL2 together with increased phagocytosis, nitric oxide production, and fungicidal activity	IL-10 suppresses an MCP-1/CCL2-associated activated macrophage response	Moderate [120]
P. brasiliensis	Lung and macrophages	TLR4-competent and TLR4-defective macrophages infected with viable yeast; TLR4-competent and TLR4-defective mice infected intratracheally; protein measured by ELISA; inflammatory-cell recruitment assessed by flow cytometry	TLR4 signaling deficiency	TLR4-defective macrophages produce less MCP-1/CCL2. TLR4-defective mice also show reduced pulmonary chemokine production and inflammatory recruitment	TLR4 promotes MCP-1/CCL2 induction and inflammatory recruitment, although stronger signaling also accompanies greater disease severity	Moderate [121]
P. brasiliensis	Macrophages	WT and MyD88$^{-/-}$ peritoneal macrophages infected with viable yeast; supernatant protein measured by ELISA	MyD88 genetic deletion	MyD88-deficient macrophages produce less MCP-1/CCL2 after fungal stimulation	MyD88 is an upstream regulator of macrophage MCP-1/CCL2 production	Moderate [122]

P. brasiliensis	Alveolar macrophages	Alveolar macrophages from susceptible B10.A and resistant A/J mice primed with IFN-γ and/or IL-12 and infected with yeast; supernatant protein measured by ELISA	Host-strain comparison and IFN-γ or IL-12 priming	Susceptible B10.A macrophages produce substantially more MCP-1/CCL2 than resistant A/J macrophages	Host genetic background strongly influences MCP-1/CCL2 production, but an MCP-1/CCL2-specific functional role is not established	Weak [123]
P. brasiliensis	Brain	Mice infected intracranially with yeast; brain MCP-1/CCL2 protein measured by ELISA at 4 and 8 weeks	Experimental CNS infection and longitudinal comparison	Brain MCP-1/CCL2 protein increases at 8 weeks together with other chemokines and progressive leukocyte recruitment	MCP-1/CCL2 is associated with later chronic CNS inflammation	Weak [124]
Paracoccidioides spp.	Serum	Adults with paracoccidioidomycosis followed before and during antifungal therapy, serial serum mediators measured by ELISA	Antifungal treatment over time	Serum MCP-1/CCL2 is low before treatment, increases at 12 months, and then declines	MCP-1/CCL2 may reflect treatment-associated immune remodeling rather than active fungal burden	Weak [125]
Coccidioides immitis	CNS and basilar arteries	Rabbit coccidioidal meningitis model, basilar-artery MCP-1/CCL2 mRNA and histopathology assessed over time	Experimental intracisternal infection and longitudinal comparison	MCP-1/CCL2 mRNA is elevated in infected basilar arteries during meningitis-associated vasculitis but does not closely track histologic severity	MCP-1/CCL2 is associated with CNS vascular inflammation without demonstrated MCP-1/CCL2-dependent function	Weak [126]
Rhizopus arrhizus, *Rhizomucor pusillus*, and *Cunninghamella bertholletiae*	Alveolar epithelial and endothelial barrier	Human Transwell bilayer model with or without monocyte-derived dendritic cells; MCP-1/CCL2 mRNA and protein assessed	Addition or absence of monocyte-derived dendritic cells	MCP-1/CCL2 responses are weak in epithelial-only conditions but increase when dendritic cells are present	MCP-1/CCL2 is part of a dendritic-cell-amplified barrier response, but its contribution to reduced fungal invasion is not directly tested	Weak [127]
Mucorales spp.	Serum and PBMCs	Patients with COVID-19-associated mucormycosis (CAM) and COVID-19	Ex vivo CotH3 peptide stimulation and	CAM patients show higher serum MCP-1/CCL2 and stronger MCP-1/CCL2 release	MCP-1/CCL2 is part of systemic inflammatory dysregulation, but host-risk	Weak [128]

		controls; serum protein measured by Bio-Plex Pro/Luminex multiplex assay; PBMCs stimulated ex vivo with CotH3 peptide and supernatant cytokines measured	clinical subgroup comparison	after CotH3 stimulation. Levels are also higher with diabetes and corticosteroid exposure	factors confound attribution specifically to mucormycosis	
Stachybotrys chartarum	Lung	BALB/c and C57BL/6 mice exposed intranasally to fungal spores; BALF chemokine protein measured by multi-analyte protein profiling	Mouse-strain comparison	MCP-family chemokines increase after exposure, with stronger responses in BALB/c mice	MCP-1/CCL2 is part of strain-dependent mold-induced pulmonary inflammation	Weak [129]
S. chartarum	Lung	BALB/c mice repeatedly exposed to satratoxin-producing or nonproducing fungal spores; lung mRNA measured by quantitative RT-PCR	Comparison of satratoxin-producing and nonproducing strains	MCP-1/CCL2 mRNA increases after repeated exposure to both fungal strains	Mold-induced pulmonary MCP-1/CCL2 does not depend strictly on satratoxin production	Weak [130]
Bjerkandera adusta	Lung and macrophages	WT, TLR-deficient, and MyD88-deficient mice repeatedly challenged with *B. adusta*; BMDMs stimulated in vitro; BALF protein measured by ELISA	TLR and MyD88 genetic deletion	MCP-1/CCL2 induction is reduced in MyD88-deficient mice and in selected MyD88 or TLR-deficient macrophage conditions	Innate fungal-recognition pathways involving MyD88 regulate MCP-1/CCL2 production	Moderate [131]
Fusarium solani	Cornea	Ex vivo goat corneas infected with a clinical *F. solani* isolate; culture-supernatant protein measured by ELISA at serial time points	Posaconazole, voriconazole, natamycin, or amphotericin B treatment	MCP-1/CCL2 remains elevated during infection and decreases after treatment, even when fungal hyphae remain detectable under some conditions	MCP-1/CCL2 reflects local corneal inflammation and treatment response more closely than complete fungal eradication	Weak [132]
Trichophyton mentagrophytes, *T.*	Keratinocytes	Human epidermal keratinocytes exposed to dermatophytes for 3 to 24 h;	Fungal exposure only	MCP-1/CCL2 remains low and does not significantly increase during early fungal exposure	MCP-1/CCL2 is not a prominent component of the early keratinocyte response to dermatophytes	Weak [133]

tonsurans, and *T. rubrum*		secreted MCP-1/CCL2 protein measured by ELISA				
Malassezia sympodialis	Mast cells	WT, TLR2−/−, TLR4−/−, and MyD88−/− bone marrow-derived mast cells stimulated with extract; protein release measured by ELISA	TLR2, TLR4, or MyD88 genetic deletion	*M. sympodialis* induces MCP-1/CCL2 in IgE-sensitized mast cells and the response persists despite MyD88 deficiency	MCP-1/CCL2 induction occurs through a pathway largely independent of TLR2/MyD88 signaling	Moderate [134]
Pseudogymnoascus destructans	Bat wing	Naturally infected and control bat wing tissue; fungal infection confirmed by histopathology and *P. destructans*-specific qPCR; host CCL2 transcription measured by RNA-seq	Natural infection comparison	MCP-1/CCL2 transcripts increase in infected wing tissue despite limited effective leukocyte infiltration	Local MCP-1/CCL2 transcription accompanies antifungal signaling but does not demonstrate effective MCP-1/CCL2-dependent recruitment	Weak [135]
Saccharomyces cerevisiae	Peritoneal cavity and macrophages	*S. cerevisiae*-infected mice and macrophage signaling models; MCP-1/CCL2, CCL7, and CCL12 mRNA/protein measured by qRT-PCR/ELISA; inflammatory-monocyte recruitment assessed by flow cytometry; NFATc1 binding to the CCL2 promoter assessed by ChIP$	Calcineurin inhibition, NFATc1 deficiency, and constitutively active NFATc1	Calcineurin-NFATc1 signaling induces MCP-1/CCL2, CCL7, and CCL12. NFATc1 relieves BCL6-mediated repression at the MCP-1/CCL2 promoter	Defines a molecular pathway controlling MCP-1/CCL2 and other CCR2 ligands during fungal recognition and monocyte recruitment	Moderate [136]

*RPA = RNase protection assay

*TRIF = Toll/Interleukin-1 receptor domain (TIR)-domain-containing adapter-inducing interferon-β

#CRP = C-reactive protein

##LDH = Lactate dehydrogenase

$ChIP = Chromatin immunoprecipitation

Evidence strength refers to support for an MCP-1/CCL2-specific biological role rather than overall study quality. Strong evidence includes direct MCP-1/CCL2 manipulation with functional outcomes; moderate evidence includes receptor, pathway, cellular, or fungal-factor manipulation linked to MCP-1/CCL2 and a biological outcome; weak evidence is primarily associative or descriptive without direct demonstration of MCP-1/CCL2 function.

1. Huffnagle, G.B.; Strieter, R.M.; Standiford, T.J.; McDonald, R.A.; Burdick, M.D.; Kunkel, S.L.; Toews, G.B. The role of monocyte chemotactic protein-1 (MCP-1) in the recruitment of monocytes and CD4+ T cells during a pulmonary Cryptococcus neoformans infection. *The Journal of Immunology* **1995**, *155*, 4790–4797.
2. Kawakami, K.; Kinjo, Y.; Uezu, K.; Yara, S.; Miyagi, K.; Koguchi, Y.; Nakayama, T.; Taniguchi, M.; Saito, A. Monocyte Chemoattractant Protein-1-Dependent Increase of Vα14 NKT Cells in Lungs and Their Roles in Th1 Response and Host Defense in Cryptococcal Infection1. *The Journal of Immunology* **2001**, *167*, 6525–6532, doi:10.4049/jimmunol.167.11.6525.
3. Uezu, K.; Kawakami, K.; Miyagi, K.; Kinjo, Y.; Kinjo, T.; Ishikawa, H.; Saito, A. Accumulation of γδ T Cells in the Lungs and Their Regulatory Roles in Th1 Response and Host Defense against Pulmonary Infection with Cryptococcus neoformans1. *The Journal of Immunology* **2004**, *172*, 7629–7634, doi:10.4049/jimmunol.172.12.7629.
4. Traynor, T.R.; Kuziel, W.A.; Toews, G.B.; Huffnagle, G.B. CCR2 Expression Determines T1 Versus T2 Polarization During Pulmonary Cryptococcus neoformans Infection1. *The Journal of Immunology* **2000**, *164*, 2021–2027, doi:10.4049/jimmunol.164.4.2021.
5. Traynor, T.R.; Herring, A.C.; Dorf, M.E.; Kuziel, W.A.; Toews, G.B.; Huffnagle, G.B. Differential Roles of CC Chemokine Ligand 2/Monocyte Chemotactic Protein-1 and CCR2 in the Development of T1 Immunity1. *The Journal of Immunology* **2002**, *168*, 4659–4666, doi:10.4049/jimmunol.168.9.4659.
6. Osterholzer, J.J.; Curtis, J.L.; Polak, T.; Ames, T.; Chen, G.-H.; McDonald, R.; Huffnagle, G.B.; Toews, G.B. CCR2 Mediates Conventional Dendritic Cell Recruitment and the Formation of Bronchovascular Mononuclear Cell Infiltrates in the Lungs of Mice Infected with Cryptococcus neoformans1,2. *The Journal of Immunology* **2008**, *181*, 610–620, doi:10.4049/jimmunol.181.1.610.
7. Rivera, J.; Zaragoza, O.; Casadevall, A. Antibody-Mediated Protection against <i>Cryptococcus neoformans</i> Pulmonary Infection Is Dependent on B Cells. *Infection and Immunity* **2005**, *73*, 1141–1150, doi:doi:10.1128/iai.73.2.1141-1150.2005.
8. Mednick, A.J.; Feldmesser, M.; Rivera, J.; Casadevall, A. Neutropenia alters lung cytokine production in mice and reduces their susceptibility to pulmonary cryptococcosis. *European journal of immunology* **2003**, *33*, 1744–1753.
9. Kawakami, K.; Qureshi, M.H.; Zhang, T.; Koguchi, Y.; Shibuya, K.; Naoe, S.; Saito, A. Interferon-gamma (IFN-gamma)-dependent protection and synthesis of chemoattractants for mononuclear leucocytes caused by IL-12 in the lungs of mice infected with Cryptococcus neoformans. *Clin Exp Immunol* **1999**, *117*, 113–122, doi:10.1046/j.1365-2249.1999.00955.x.
10. Kawakami, K.; Shibuya, K.; Qureshi, M.H.; Zhang, T.; Koguchi, Y.; Tohyama, M.; Xie, Q.; Naoe, S.; Saito, A. Chemokine responses and accumulation of inflammatory cells in the lungs of mice infected with highly virulent Cryptococcus neoformans: effects of interleukin-12. *FEMS Immunology & Medical Microbiology* **1999**, *25*, 391–402, doi:10.1111/j.1574-695X.1999.tb01365.x.
11. Herring, A.C.; Falkowski, N.R.; Chen, G.-H.; McDonald, R.A.; Toews, G.B.; Huffnagle, G.B. Transient Neutralization of Tumor Necrosis Factor Alpha Can Produce a Chronic Fungal Infection in an Immunocompetent Host: Potential Role of Immature Dendritic Cells. *Infection and Immunity* **2005**, *73*, 39–49, doi:doi:10.1128/iai.73.1.39-49.2005.
12. He, W.; Casadevall, A.; Lee, S.C.; Goldman, D.L. Phagocytic Activity and Monocyte Chemotactic Protein Expression by Pulmonary Macrophages in Persistent Pulmonary Cryptococcosis. *Infection and Immunity* **2003**, *71*, 930–936, doi:doi:10.1128/iai.71.2.930-936.2003.

13. Castro-Lopez, N.; Campuzano, A.; Mdalel, E.; Vanegas, D.; Chaturvedi, A.; Nguyen, P.; Pulse, M.; Cardona Astrid, E.; Wormley Floyd, L. Inhibition of host 5-lipoxygenase reduces overexuberant inflammatory responses and mortality associated with Cryptococcus meningoencephalitis. *mBio* **2024**, *15*, e01483–01424, doi:10.1128/mbio.01483-24.
14. Levitz, S.M.; North, E.A.; Jiang, Y.; Nong, S.H.; Kornfeld, H.; Harrison, T.S. Variables affecting production of monocyte chemotactic factor 1 from human leukocytes stimulated with Cryptococcus neoformans. *Infection and Immunity* **1997**, *65*, 903–908, doi:doi:10.1128/iai.65.3.903-908.1997.
15. Lopes, J.P.; Stylianou, M.; Backman, E.; Holmberg, S.; Ekoff, M.; Nilsson, G.; Urban, C.F. Cryptococcus neoformans Induces MCP-1 Release and Delays the Death of Human Mast Cells. *Frontiers in Cellular and Infection Microbiology* **2019**, *Volume 9 - 2019*, doi:10.3389/fcimb.2019.00289.
16. Souza, H.I.; Pereira, A.B.M.; Oliveira, J.R.; Silva, P.R.; Teixeira, D.N.S.; Silva-Vergara, M.L.; Rogério, A.P. Cryptococcus neoformans in Association with Dermatophagoides pteronyssinus has Pro- (IL-6/STAT3 Overproduction) and Anti-inflammatory (CCL2/ERK1/2 Downregulation) Effects on Human Bronchial Epithelial Cells. *Inflammation* **2022**, *45*, 1269–1280, doi:10.1007/s10753-021-01619-4.
17. Cunha, M.M.; Pereira, A.B.M.; Lino, R.C.; da Silva, P.R.; Andrade-Silva, L.E.; de Vito, F.B.; de Souza, H.M.; Silva-Vergara, M.L.; Rogério, A.P. Effects of combination of Cryptococcus gattii and IFN-γ, IL-4 or IL-27 on human bronchial epithelial cells. *Immunobiology* **2023**, *228*, 152312, doi:https://doi.org/10.1016/j.imbio.2022.152312.
18. Mednick, A.J.; Nosanchuk, J.D.; Casadevall, A. Melanization of <i>Cryptococcus neoformans</i> Affects Lung Inflammatory Responses during Cryptococcal Infection. *Infection and Immunity* **2005**, *73*, 2012–2019, doi:doi:10.1128/iai.73.4.2012-2019.2005.
19. Wormley Jr, F.L.; Cox, G.M.; Perfect, J.R. Evaluation of host immune responses to pulmonary cryptococcosis using a temperature-sensitive C. neoformans calcineurin A mutant strain. *Microbial Pathogenesis* **2005**, *38*, 113–123, doi:https://doi.org/10.1016/j.micpath.2004.12.007.
20. Xu, J.; Flaczyk, A.; Neal, L.M.; Fa, Z.; Eastman, A.J.; Malachowski, A.N.; Cheng, D.; Moore, B.B.; Curtis, J.L.; Osterholzer, J.J.; et al. Scavenger Receptor MARCO Orchestrates Early Defenses and Contributes to Fungal Containment during Cryptococcal Infection. *The Journal of Immunology* **2017**, *198*, 3548–3557, doi:10.4049/jimmunol.1700057.
21. Supasorn, O.; Sringkarin, N.; Srimanote, P.; Angkasekwinai, P. Matrix metalloproteinases contribute to the regulation of chemokine expression and pulmonary inflammation in Cryptococcus infection. *Clinical and Experimental Immunology* **2016**, *183*, 431–440, doi:10.1111/cei.12725.
22. Farnoud, A.M.; Bryan, A.M.; Kechichian, T.; Luberto, C.; Poeta, M.D. The Granuloma Response Controlling Cryptococcosis in Mice Depends on the Sphingosine Kinase 1–Sphingosine 1-Phosphate Pathway. *Infection and Immunity* **2015**, *83*, 2705–2713, doi:doi:10.1128/iai.00056-15.
23. Grahnert, A.; Richter, T.; Piehler, D.; Eschke, M.; Schulze, B.; Müller, U.; Protschka, M.; Köhler, G.; Sabat, R.; Brombacher, F.; et al. IL-4 receptor-alpha-dependent control of Cryptococcus neoformans in the early phase of pulmonary infection. *PLoS One* **2014**, *9*, e87341, doi:10.1371/journal.pone.0087341.
24. Edwards, L.; Williams, A.E.; Krieg, A.M.; Rae, A.J.; Snelgrove, R.J.; Hussell, T. Stimulation via Toll-like receptor 9 reduces Cryptococcus neoformans-induced pulmonary inflammation in an IL-12-dependent manner. *Eur J Immunol* **2005**, *35*, 273–281, doi:10.1002/eji.200425640.
25. Arromsava, A.; Chuchawankul, S.; Worasilchai, N.; Angkasekwinai, P.; Amornsupak, K. Immunomodulatory activity of Pleurotus pulmonarius crude extract to human monocyte against Cryptococcus neoformans. *BMC Complementary Medicine and Therapies* **2025**, *25*, 251, doi:10.1186/s12906-025-04990-z.
26. Xu, J.; Ganguly, A.; Zhao, J.; Ivey, M.; Lopez, R.; Osterholzer, J.J.; Cho, C.S.; Olszewski, M.A. CCR2 Signaling Promotes Brain Infiltration of Inflammatory Monocytes and Contributes to Neuropathology during Cryptococcal Meningoencephalitis. *mBio* **2021**, *12*, 10.1128/mbio.01076–01021, doi:doi:10.1128/mbio.01076-21.

27. Shimoda, M.; Jones, V.C.; Kobayashi, M.; Suzuki, F. Microglial cells from psychologically stressed mice as an accelerator of cerebral cryptococcosis. *Immunology and cell biology* **2006**, *84*, 551–556.

28. Kleinschek, M.A.; Muller, U.; Brodie, S.J.; Stenzel, W.; Kohler, G.; Blumenschein, W.M.; Straubinger, R.K.; McClanahan, T.; Kastelein, R.A.; Alber, G. IL-23 Enhances the Inflammatory Cell Response in Cryptococcus neoformans Infection and Induces a Cytokine Pattern Distinct from IL-121. *The Journal of Immunology* **2006**, *176*, 1098–1106, doi:10.4049/jimmunol.176.2.1098.

29. Fries, B.C.; Lee, S.C.; Kennan, R.; Zhao, W.; Casadevall, A.; Goldman, D.L. Phenotypic Switching of <i>Cryptococcus neoformans</i> Can Produce Variants That Elicit Increased Intracranial Pressure in a Rat Model of Cryptococcal Meningoencephalitis. *Infection and Immunity* **2005**, *73*, 1779–1787, doi:doi:10.1128/iai.73.3.1779-1787.2005.

30. Uicker, W.C.; Doyle, H.A.; McCracken, J.P.; Langlois, M.; Buchanan, K.L. Cytokine and chemokine expression in the central nervous system associated with protective cell-mediated immunity against Cryptococcus neoformans. *Medical Mycology* **2005**, *43*, 27–38, doi:10.1080/13693780410001731510.

31. Rayhane, N.; Lortholary, O.; Fitting, C.; Callebert, J.; Huerre, M.; Dromer, F.; Cavaillon, J.-M. Enhanced Sensitivity of Tumor Necrosis Factor/Lymphotoxin-α—Deficient Mice to Cryptococcus neoformans Infection despite Increased Levels of Nitrite/Nitrate, Interferon-γ, and Interleukin-12. *The Journal of Infectious Diseases* **1999**, *180*, 1637–1647, doi:10.1086/315061.

32. Jarvis, J.N.; Meintjes, G.; Bicanic, T.; Buffa, V.; Hogan, L.; Mo, S.; Tomlinson, G.; Kropf, P.; Noursadeghi, M.; Harrison, T.S. Cerebrospinal fluid cytokine profiles predict risk of early mortality and immune reconstitution inflammatory syndrome in HIV-associated cryptococcal meningitis. *PLoS Pathog* **2015**, *11*, e1004754, doi:10.1371/journal.ppat.1004754.

33. Chang, C.C.; Omarjee, S.; Lim, A.; Spelman, T.; Gosnell, B.I.; Carr, W.H.; Elliott, J.H.; Moosa, M.-Y.S.; Ndung'u, T.; French, M.A.; et al. Chemokine Levels and Chemokine Receptor Expression in the Blood and the Cerebrospinal Fluid of HIV-Infected Patients With Cryptococcal Meningitis and Cryptococcosis-Associated Immune Reconstitution Inflammatory Syndrome. *The Journal of Infectious Diseases* **2013**, *208*, 1604–1612, doi:10.1093/infdis/jit388.

34. Zhou, L.H.; Zhao, H.Z.; Wang, X.; Wang, R.Y.; Jiang, Y.K.; Huang, L.P.; Yip, C.W.; Cheng, J.H.; Que, C.X.; Zhu, L.P. Immune reconstitution inflammatory syndrome in non-HIV cryptococcal meningitis: Cross-talk between pathogen and host. *Mycoses* **2021**, *64*, 1402–1411, doi:10.1111/myc.13361.

35. Tugume, L.; Rhein, J.; Hullsiek, K.H.; Mpoza, E.; Kiggundu, R.; Ssebambulidde, K.; Schutz, C.; Taseera, K.; Williams, D.A.; Abassi, M.; et al. HIV-Associated Cryptococcal Meningitis Occurring at Relatively Higher CD4 Counts. *The Journal of Infectious Diseases* **2019**, *219*, 877–883, doi:10.1093/infdis/jiy602.

36. Lofgren, S.; Hullsiek, K.H.; Morawski, B.M.; Nabeta, H.W.; Kiggundu, R.; Taseera, K.; Musubire, A.; Schutz, C.; Abassi, M.; Bahr, N.C.; et al. Differences in Immunologic Factors Among Patients Presenting with Altered Mental Status During Cryptococcal Meningitis. *The Journal of Infectious Diseases* **2017**, *215*, 693–697, doi:10.1093/infdis/jix033.

37. Xu, L.; Xu, Y.; Zheng, Y.; Peng, X.; Yang, Z.; Cao, Q.; Xiang, D.; Zhao, H. Differences in cytokine and chemokine profiles in cerebrospinal fluid caused by the etiology of cryptococcal meningitis and tuberculous meningitis in HIV patients. *Clinical and Experimental Immunology* **2021**, *206*, 82–90, doi:10.1111/cei.13644.

38. Christo, P.P.; de Carvalho Vilela, M.; Bretas, T.L.; Domingues, R.B.; Greco, D.B.; Livramento, J.A.; Teixeira, A.L. Cerebrospinal fluid levels of chemokines in HIV infected patients with and without opportunistic infection of the central nervous system. *Journal of the neurological sciences* **2009**, *287*, 79–83.

39. Li, A.; Zhu, W.; Yin, J.; Huang, X.; Sun, L.; Hua, W.; Wang, W.; Zhang, T.; Dai, L.; Wu, H. A preliminary study on the characteristics of Th1/Th2 immune response in cerebrospinal fluid of AIDS patients with cryptococcal meningitis. *BMC Infectious Diseases* **2021**, *21*, 500, doi:10.1186/s12879-021-06138-z.
40. Beardsley, J.; Hoang, N.L.T.; Kibengo, F.M.; Tung, N.L.N.; Binh, T.Q.; Hung, L.Q.; Chierakul, W.; Thwaites, G.E.; Chau, N.V.V.; Nguyen, T.T.T.; et al. Do Intracerebral Cytokine Responses Explain the Harmful Effects of Dexamethasone in Human Immunodeficiency Virus–associated Cryptococcal Meningitis? *Clinical Infectious Diseases* **2019**, *68*, 1494–1501, doi:10.1093/cid/ciy725.
41. Wu, L.; Fu, X.; Pütz, B.; Zhang, R.; Liu, L.; Song, W.; Weng, L.; Shao, Y.; Zheng, Z.; Xun, J.; et al. Comprehensive risk factor predictions for 3-year survival among HIV-associated and disseminated cryptococcosis involving lungs and central nervous system. *Infection* **2024**, *52*, 1875–1887, doi:10.1007/s15010-024-02237-6.
42. Saavedra, M.; Taylor, B.; Lukacs, N.; Fidel, P.L. Local Production of Chemokines during Experimental Vaginal Candidiasis. *Infection and Immunity* **1999**, *67*, 5820–5826, doi:doi:10.1128/iai.67.11.5820-5826.1999.
43. Steele, C.; Fidel, P.L. Cytokine and Chemokine Production by Human Oral and Vaginal Epithelial Cells in Response to <i>Candida albicans</i>. *Infection and Immunity* **2002**, *70*, 577–583, doi:doi:10.1128/iai.70.2.577-583.2002.
44. Kuboyama, N.; Hayakawa, E.; Yaguchi, M.; Nomoto, T.; Abiko, Y. Stimulation of CCL2 Expression in Human Gingival Epithelium <i>by Candida albicans </i>. *International Journal of Oral-Medical Sciences* **2012**, *11*, 90–95, doi:10.5466/ijoms.11.90.
45. Zeise, K.D.; Falkowski, N.R.; Metcalf, J.D.; Brown, C.A.; Huffnagle, G.B. Gene expression profiling reveals host defense strategies for restricting <i>Candida albicans</i> invasion and gastritis to the limiting ridge of the murine stomach. *Infection and Immunity* **2024**, *92*, e00438–00424, doi:doi:10.1128/iai.00438-24.
46. MuÃ Â±oz-Duarte, A.R.; CastrejÃ Â³n-JimÃ Â©nez, N.S.; Baltierra-Uribe, S.L.; PÃ Â©rez-Rangel, S.J.; Carapia-Minero, N.; CastaÃ Â±eda-SÃ Â¡nchez, J.I.; Luna-Herrera, J.; LÃ Â³pez-Santiago, R.Â.n.; RodrÃ Â-guez-Tovar, A.Â.d.V.Â.n.; GarcÃ Â-a-PÃ Â©rez, B.E. Candida glabrata survives and replicates in human osteoblasts. *Pathogens and Disease* **2016**, *74*, doi:10.1093/femspd/ftw030.
47. Xu, T.; Liu, Y.; Zhang, W.; Li, M.; Zhang, L.; Li, X.; Zhang, Y.; Yue, L.; Li, S.; Lin, Y.; et al. Specific cell subclusters of dental pulp stem cells respond to distinct pathogens through the ROS pathway. *Frontiers in Cellular and Infection Microbiology* **2024**, *Volume 14 - 2024*, doi:10.3389/fcimb.2024.1452124.
48. Jakkawanpitak, C.; Hutadilok-Towatana, N.; Sermwittayawong, D. Fungal-like particles and macrophage-conditioned medium are inflammatory elicitors for 3T3-L1 adipocytes. *Scientific Reports* **2020**, *10*, 9437, doi:10.1038/s41598-020-66283-4.
49. Saunus, J.M.; Wagner, S.A.; Matias, M.A.; Hu, Y.; Zaini, Z.M.; Farah, C.S. Early activation of the interleukin-23-17 axis in a murine model of oropharyngeal candidiasis. *Mol Oral Microbiol* **2010**, *25*, 343–356, doi:10.1111/j.2041-1014.2010.00570.x.
50. Castillo, L.; MacCallum, D.M.; Brown, A.J.P.; Gow, N.A.R.; Odds, F.C. Differential Regulation of Kidney and Spleen Cytokine Responses in Mice Challenged with Pathology-Standardized Doses of <i>Candida albicans</i> Mannosylation Mutants. *Infection and Immunity* **2011**, *79*, 146–152, doi:doi:10.1128/iai.01004-10.
51. Villar, C.C.; Kashleva, H.; Mitchell, A.P.; Dongari-Bagtzoglou, A. Invasive Phenotype of <i>Candida albicans</i> Affects the Host Proinflammatory Response to Infection. *Infection and Immunity* **2005**, *73*, 4588–4595, doi:doi:10.1128/iai.73.8.4588-4595.2005.

52. Swidergall, M.; Khalaji, M.; Solis, N.V.; Moyes, D.L.; Drummond, R.A.; Hube, B.; Lionakis, M.S.; Murdoch, C.; Filler, S.G.; Naglik, J.R. Candidalysin Is Required for Neutrophil Recruitment and Virulence During Systemic Candida albicans Infection. *The Journal of Infectious Diseases* **2019**, *220*, 1477–1488, doi:10.1093/infdis/jiz322.
53. Miyabe, C.; Miyabe, Y.; Bricio-Moreno, L.; Lian, J.; Rahimi, R.A.; Miura, N.N.; Ohno, N.; Iwakura, Y.; Kawakami, T.; Luster, A.D. Dectin-2-induced CCL2 production in tissue-resident macrophages ignites cardiac arteritis. *J Clin Invest* **2019**, *129*, 3610–3624, doi:10.1172/jci123778.
54. Liu, S.; Lin, K.; Tian, Y.; He, Y.; Zhang, Z.; Li, C.; Luo, Y.; Liang, Y.; Ma, Y.; Deng, W. Candidalysin induces Tim-3 expression in macrophages to suppress antifungal immunity in oropharyngeal candidiasis. *International Immunopharmacology* **2025**, *156*, 114672, doi:https://doi.org/10.1016/j.intimp.2025.114672.
55. Kobbe, R.; Kolster, M.; Fuchs, S.; Schulze-Sturm, U.; Jenderny, J.; Kochhan, L.; Staab, J.; Tolosa, E.; Grimbacher, B.; Meyer, T. Common variable immunodeficiency, impaired neurological development and reduced numbers of T regulatory cells in a 10-year-old boy with a STAT1 gain-of-function mutation. *Gene* **2016**, *586*, 234–238, doi:https://doi.org/10.1016/j.gene.2016.04.006.
56. Tan, W.; Liu, J.; Yu, R.; Zhao, P.; Liu, Y.; Lu, Q.; Wang, K.; Ding, H.; Liu, Y.; Lai, X.; et al. Trim72 is a major host factor protecting against lethal Candida albicans infection. *PLoS Pathog* **2024**, *20*, e1012747, doi:10.1371/journal.ppat.1012747.
57. Majer, O.; Bourgeois, C.; Zwolanek, F.; Lassnig, C.; Kerjaschki, D.; Mack, M.; Müller, M.; Kuchler, K. Type I Interferons Promote Fatal Immunopathology by Regulating Inflammatory Monocytes and Neutrophils during Candida Infections. *PLOS Pathogens* **2012**, *8*, e1002811, doi:10.1371/journal.ppat.1002811.
58. de Vries, D.H.; Matzaraki, V.; Bakker, O.B.; Brugge, H.; Westra, H.J.; Netea, M.G.; Franke, L.; Kumar, V.; van der Wijst, M.G.P. Integrating GWAS with bulk and single-cell RNA-sequencing reveals a role for LY86 in the anti-Candida host response. *PLoS Pathog* **2020**, *16*, e1008408, doi:10.1371/journal.ppat.1008408.
59. Venturini, J.; Fraga-Silva, T.F.; Marchetti, C.M.; Mimura, L.A.; Conti, B.J.; Golim Mde, A.; Mendes, R.P.; de Arruda, M.S. Imbalanced Macrophage and Dendritic Cell Activations in Response to Candida albicans in a Murine Model of Diabetes Mellitus. *Immunol Invest* **2016**, *45*, 420–438, doi:10.3109/08820139.2016.1162798.
60. Pinke, K.H.; Freitas, P.; Viera, N.A.; Honório, H.M.; Porto, V.C.; Lara, V.S. Decreased production of proinflammatory cytokines by monocytes from individuals presenting Candida-associated denture stomatitis. *Cytokine* **2016**, *77*, 145–151, doi:https://doi.org/10.1016/j.cyto.2015.10.017.
61. Righi, S.E.; Lilly, E.A.; Harriett, A.J.; Daly, P.W.; Jones, M.; Steele, C.; Noverr, M.C.; Fidel, P.L., Jr. Induction of myelopoiesis by Candida dubliniensis drives protective trained immunity against sepsis in a Card9-dependent manner. *mBio* **2025**, *16*, e0290625, doi:10.1128/mbio.02906-25.
62. Radovanovic, I.; Leung, V.; Iliescu, A.; Bongfen, S.E.; Mullick, A.; Langlais, D.; Gros, P. Genetic Control of Susceptibility to Candida albicans in SM/J Mice. *The Journal of Immunology* **2014**, *193*, 1290–1300, doi:10.4049/jimmunol.1400783.
63. MacCallum, D.M.; Castillo, L.; Brown, A.J.; Gow, N.A.; Odds, F.C. Early-expressed chemokines predict kidney immunopathology in experimental disseminated Candida albicans infections. *PLoS One* **2009**, *4*, e6420, doi:10.1371/journal.pone.0006420.
64. Mullick, A.; Elias, M.; Picard, S.; Bourget, L.; Jovcevski, O.; Gauthier, S.; Tuite, A.; Harakidas, P.; Bihun, C.; Massie, B.; et al. Dysregulated Inflammatory Response to <i>Candida albicans</i> in a C5-Deficient Mouse Strain. *Infection and Immunity* **2004**, *72*, 5868–5876, doi:doi:10.1128/iai.72.10.5868-5876.2004.
65. Allen, B.; Dambuza, I.M.; Berry, S.H.; Reid, D.M.; McVey, S.M.; Mesiarikova, M.; John, L.; Davie, M.; Baker, C.P.; Arthur, J.S.C.; et al. PTP1B deficiency in myeloid cells increases susceptibility to <i>Candida albicans</i> systemic infection by modulating antifungal immunity. *mBio* **2025**, *16*, e01516–01525, doi:doi:10.1128/mbio.01516-25.

66. Gander-Bui, H.T.T.; Schläfli, J.; Baumgartner, J.; Walthert, S.; Genitsch, V.; van Geest, G.; Galván, J.A.; Cardozo, C.; Graham Martinez, C.; Grans, M.; et al. Targeted removal of macrophage-secreted interleukin-1 receptor antagonist protects against lethal Candida albicans sepsis. *Immunity* **2023**, *56*, 1743–1760.e1749, doi:10.1016/j.immuni.2023.06.023.
67. Teo, Y.J.; Ng, S.L.; Mak, K.W.; Setiagani, Y.A.; Chen, Q.; Nair, S.K.; Sheng, J.; Ruedl, C. Renal CD169(++) resident macrophages are crucial for protection against acute systemic candidiasis. *Life Sci Alliance* **2021**, *4*, doi:10.26508/lsa.202000890.
68. Zhang, H.; Li, H.; Li, Y.; Zou, Y.; Dong, X.; Song, W.; Jia, C.; Li, S.; Xi, H.; Liu, D.; et al. IL-17 plays a central role in initiating experimental Candida albicans infection in mouse corneas. *Eur J Immunol* **2013**, *43*, 2671–2682, doi:10.1002/eji.201242891.
69. Freitas, C.G.; Lima, S.M.F.; Freire, M.S.; Cantuária, A.P.C.; Júnior, N.G.O.; Santos, T.S.; Folha, J.S.; Ribeiro, S.M.; Dias, S.C.; Rezende, T.M.B.; et al. An Immunomodulatory Peptide Confers Protection in an Experimental Candidemia Murine Model. *Antimicrobial Agents and Chemotherapy* **2017**, *61*, 10.1128/aac.02518–02516, doi:doi:10.1128/aac.02518-16.
70. Xie, Y.; Wang, R.; Wu, Z.; Xie, C.; Gong, S.; Zhang, J.; Yu, H.; Song, Z. Prophylactic application of sodium new houttuyfonate to regulate macrophage activation and antifungal infection in intra-abdominal candidiasis model mice. *International immunopharmacology* **2025**, *159*, 114922.
71. Jordão, C.C.; Klein, M.I.; Barbugli, P.A.; Ferrisse, T.M.; de Moraes, J.C.G.; Pavarina, A.C. The association of DNase I with antimicrobial photodynamic therapy affects Candida albicans gene expression and promotes immunomodulatory effects in mice with candidiasis. *Photochemical & Photobiological Sciences* **2025**, *24*, 791–802, doi:10.1007/s43630-025-00727-6.
72. Yang, D.L.; Zhang, Y.Q.; Hu, Y.L.; Weng, L.X.; Zeng, G.S.; Wang, L.H. Protective effects of cis-2-dodecenoic acid in an experimental mouse model of vaginal candidiasis. *Biomedical and Environmental Sciences* **2018**, *31*, 816–828.
73. Li, X.Y.; Zhang, Y.Q.; Xu, G.; Li, S.H.; Li, H. miR-124/MCP-1 signaling pathway modulates the protective effect of itraconazole on acute kidney injury in a mouse model of disseminated candidiasis. *Int J Mol Med* **2018**, *41*, 3468–3476, doi:10.3892/ijmm.2018.3564.
74. Wozniak, K.L.; Palmer, G.; Kutner, R.; Fidel, P.L., Jr. Immunotherapeutic approaches to enhance protective immunity against Candida vaginitis. *Medical Mycology* **2005**, *43*, 589–601, doi:10.1080/13693780500096898.
75. Camaioni, L.; Ustyanowski, B.; Buisine, M.; Lambert, D.; Sendid, B.; Billamboz, M.; Jawhara, S. Natural compounds with antifungal properties against Candida albicans and identification of Hinokitiol as a promising antifungal drug. *Antibiotics* **2023**, *12*, 1603.
76. Yu, F.; Jiang, W.; Zhang, L.; Jiang, N. IDO regulates macrophage functions by inhibiting the CCL2/CCR2 signaling pathway in fungal keratitis. *Cornea* **2023**, *42*, 1005–1015.
77. Xu, Q.; Zhao, G.; Lin, J.; Wang, Q.; Hu, L.; Jiang, Z. Role of Dectin-1 in the innate immune response of rat corneal epithelial cells to Aspergillus fumigatus. *BMC Ophthalmology* **2015**, *15*, 126, doi:10.1186/s12886-015-0112-1.
78. Zhao, G.; Xu, Q.; Lin, J.; Chen, W.; Cui, T.; Hu, L.; Jiang, N. The role of Mincle in innate immune to fungal keratitis. *The Journal of Infection in Developing Countries* **2017**, *11*, 89–97.
79. Yu, B.; Wang, Q.; Zhang, L.; Lin, J.; Feng, Z.; Wang, Z.; Gu, L.; Tian, X.; Luan, S.; Li, C.; et al. Ebselen improves fungal keratitis through exerting anti-inflammation, anti-oxidative stress, and antifungal effects. *Redox Biology* **2024**, *73*, 103206, doi:https://doi.org/10.1016/j.redox.2024.103206.

80. Luan, J.; Lu, Y.; Wang, Q.; Li, C.; Peng, X.; Zheng, H.; Zhang, S.; Zhao, G.; Lin, J. Sanguinarine attenuates Aspergillus fumigatus keratitis by exerting antifungal and anti-inflammatory effects. *Experimental Eye Research* **2026**, *262*, 110744, doi:https://doi.org/10.1016/j.exer.2025.110744.
81. Liu, X.; Sui, J.; Qi, P.; Luan, J.; Wang, Y.; Li, C.; Wang, Q.; Peng, X.; Zhao, G.; Lin, J. Anti-inflammatory efficacy of nanobody specific to β-glucan on a fungal cell wall in a murine model of fungal keratitis. *ACS Infectious Diseases* **2024**, *10*, 2991–2998.
82. Yin, M.; Li, N.; Zhang, L.; Lin, J.; Wang, Q.; Gu, L.; Zheng, H.; Zhao, G.; Li, C. Pseudolaric acid B ameliorates fungal keratitis progression by suppressing inflammation and reducing fungal load. *ACS Infectious Diseases* **2023**, *9*, 1196–1205.
83. He, M.; You, J.; Liu, X.; Peng, X.; Li, C.; Yang, S.; Xu, Q.; Lin, J.; Zhao, G. Perillaldehyde protects against Aspergillus fumigatus keratitis by reducing fungal load and inhibiting inflammatory cytokines and LOX-1. *Current eye research* **2022**, *47*, 1366–1373.
84. Clemons, K.V.; Danielson, M.E.; Michel, K.S.; Liu, M.; Ottoson, N.C.; Leonardo, S.M.; Martinez, M.; Chen, V.; Antonysamy, M.A.; Stevens, D.A. Whole glucan particles as a vaccine against murine aspergillosis. *Journal of medical microbiology* **2014**, *63*, 1750–1759.
85. Pérez-Cantero, A.; Serrano, D.R.; Navarro-Rodríguez, P.; Schätzlein, A.G.; Uchegbu, I.F.; Torrado, J.J.; Capilla, J. Increased efficacy of oral fixed-dose combination of amphotericin B and AHCC® natural adjuvant against aspergillosis. *Pharmaceutics* **2019**, *11*, 456.
86. Morrison, B.E.; Park, S.J.; Mooney, J.M.; Mehrad, B. Chemokine-mediated recruitment of NK cells is a critical host defense mechanism in invasive aspergillosis. *The Journal of clinical investigation* **2003**, *112*, 1862–1870.
87. Park, S.J.; Burdick, M.D.; Brix, W.K.; Stoler, M.H.; Askew, D.S.; Strieter, R.M.; Mehrad, B. Neutropenia enhances lung dendritic cell recruitment in response to Aspergillus via a cytokine-to-chemokine amplification loop. *The Journal of Immunology* **2010**, *185*, 6190–6197.
88. Carpenter, K.J.; Hogaboam, C.M. Immunosuppressive Effects of CCL17 on Pulmonary Antifungal Responses during Pulmonary Invasive Aspergillosis. *Infection and Immunity* **2005**, *73*, 7198–7207, doi:doi:10.1128/iai.73.11.7198-7207.2005.
89. Herbst, S.; Shah, A.; Carby, M.; Chusney, G.; Kikkeri, N.; Dorling, A.; Bignell, E.; Shaunak, S.; Armstrong-James, D. A new and clinically relevant murine model of solid-organ transplant aspergillosis. *Disease models & mechanisms* **2013**, *6*, 643–651.
90. Gessner, M.A.; Doran, S.F.; Yu, Z.; Dunaway, C.W.; Matalon, S.; Steele, C. Chlorine gas exposure increases susceptibility to invasive lung fungal infection. *American Journal of Physiology-Lung Cellular and Molecular Physiology* **2013**, *304*, L765–L773, doi:10.1152/ajplung.00030.2013.
91. Becker, M.J.; de Marie, S.; Fens, M.H.A.M.; Verbrugh, H.A.; Bakker-Woudenberg, I.A.J.M. Effect of amphotericin B treatment on kinetics of cytokines and parameters of fungal load in neutropenic rats with invasive pulmonary aspergillosis. *Journal of Antimicrobial Chemotherapy* **2003**, *52*, 428–434, doi:10.1093/jac/dkg367.
92. Blease, K.; Mehrad, B.; Lukacs, N.W.; Kunkel, S.L.; Standiford, T.J.; Hogaboam, C.M. Antifungal and airway remodeling roles for murine monocyte chemoattractant protein-1/CCL2 during pulmonary exposure to Asperigillus fumigatus conidia. *J Immunol* **2001**, *166*, 1832–1842, doi:10.4049/jimmunol.166.3.1832.
93. Blease, K.; Mehrad, B.; Standiford, T.J.; Lukacs, N.W.; Gosling, J.; Boring, L.; Charo, I.F.; Kunkel, S.L.; Hogaboam, C.M. Enhanced pulmonary allergic responses to Aspergillus in CCR2−/− mice. *The Journal of Immunology* **2000**, *165*, 2603–2611.

94. Koth, L.L.; Rodriguez, M.W.; Bernstein, L.X.; Chan, S.; Huang, X.; Charo, I.F.; Rollins, B.J.; Erle, D.J. Aspergillus antigen induces robust Th2 cytokine production, inflammation, airway hyperreactivity and fibrosis in the absence of MCP-1 or CCR2. *Respiratory Research* **2004**, *5*, 12, doi:10.1186/1465-9921-5-12.

95. Blease, K.; Schuh, J.M.; Jakubzick, C.; Lukacs, N.W.; Kunkel, S.L.; Joshi, B.H.; Puri, R.K.; Kaplan, M.H.; Hogaboam, C.M. Stat6-Deficient Mice Develop Airway Hyperresponsiveness and Peribronchial Fibrosis during Chronic Fungal Asthma. *The American Journal of Pathology* **2002**, *160*, 481–490, doi:10.1016/S0002-9440(10)64867-5.

96. Overton, N.; Simpson, A.; Bowyer, P.; Denning, D.W. Genetic susceptibility to severe asthma with fungal sensitization. *International Journal of Immunogenetics* **2017**, *44*, 93–106.

97. Peeters, D.; Peters, I.R.; Clercx, C.; Day, M.J. Quantification of mRNA encoding cytokines and chemokines in nasal biopsies from dogs with sino-nasal aspergillosis. *Veterinary microbiology* **2006**, *114*, 318–326.

98. Ayers, C.M.; Schlosser, R.J.; O'Connell, B.P.; Atkinson, C.; Mulligan, R.M.; Casey, S.E.; Bleier, B.S.; Wang, E.W.; Sansoni, E.R.; Kuhlen, J.L. Increased presence of dendritic cells and dendritic cell chemokines in the sinus mucosa of chronic rhinosinusitis with nasal polyps and allergic fungal rhinosinusitis. In Proceedings of the International forum of allergy & rhinology, 2011; pp. 296–302.

99. Fageräng, B.; Götz, M.P.; Cyranka, L.; Lau, C.; Nilsson, P.H.; Mollnes, T.E.; Garred, P. The Inflammatory Response Induced by Aspergillus fumigatus Conidia Is Dependent on Complement Activation: Insight from a Whole Blood Model. *J Innate Immun* **2024**, *16*, 324–336, doi:10.1159/000539368.

100. Hoang, T.N.M.; Cseresnyés, Z.; Hartung, S.; Blickensdorf, M.; Saffer, C.; Rennert, K.; Mosig, A.S.; von Lilienfeld-Toal, M.; Figge, M.T. Invasive aspergillosis-on-chip: A quantitative treatment study of human Aspergillus fumigatus infection. *Biomaterials* **2022**, *283*, 121420, doi:https://doi.org/10.1016/j.biomaterials.2022.121420.

101. Kim, Y.H.; Lee, S.-H. Mitochondrial reactive oxygen species regulate fungal protease-induced inflammatory responses. *Toxicology* **2017**, *378*, 86–94, doi:https://doi.org/10.1016/j.tox.2017.01.008.

102. Bendíčková, K.; Tidu, F.; De Zuani, M.; Kohoutková, M.H.; Andrejčinová, I.; Pompeiano, A.; Bělášková, S.; Forte, G.; Zelante, T.; Frič, J. Calcineurin inhibitors reduce NFAT-dependent expression of antifungal pentraxin-3 by human monocytes. *Journal of Leukocyte Biology* **2020**, *107*, 497–508, doi:10.1002/jlb.4vma0318-138r.

103. Vymazal, O.; Papatheodorou, I.; Andrejčinová, I.; Bosáková, V.; Vascelli, G.; Bendíčková, K.; Zelante, T.; Hortová-Kohoutková, M.; Frič, J. Calcineurin-NFAT signaling controls neutrophils' ability of chemoattraction upon fungal infection. *Journal of Leukocyte Biology* **2024**, *116*, 816–829, doi:10.1093/jleuko/qiae091.

104. Ceesay, M.M.; Kordasti, S.; Rufaie, E.; Lea, N.; Smith, M.; Wade, J.; Douiri, A.; Mufti, G.J.; Pagliuca, A. Baseline cytokine profiling identifies novel risk factors for invasive fungal disease among haematology patients undergoing intensive chemotherapy or haematopoietic stem cell transplantation. *Journal of Infection* **2016**, *73*, 280–288.

105. Sainz, J.; Lupiáñez, C.B.; Segura-Catena, J.; Vazquez, L.; Ríos, R.; Oyonarte, S.; Hemminki, K.; Försti, A.; Jurado, M. Dectin-1 and DC-SIGN polymorphisms associated with invasive pulmonary Aspergillosis infection. *PloS one* **2012**, *7*, e32273.

106. Skonieczna, K.; Styczyński, J.; Krenska, A.; Stawiński, P.; Płoski, R.; Derwich, K.; Badowska, W.; Wysocki, M.; Grzybowski, T. Massively parallel targeted resequencing reveals novel genetic variants associated with aspergillosis in paediatric patients with haematological malignancies. *Polish Journal of Pathology* **2017**, *68*, 210–217.

107. Wang, J.; Gigliotti, F.; Bhagwat, S.P.; Maggirwar, S.B.; Wright, T.W. Pneumocystis stimulates MCP-1 production by alveolar epithelial cells through a JNK-dependent mechanism. *American Journal of Physiology-Lung Cellular and Molecular Physiology* **2007**, *292*, L1495–L1505, doi:10.1152/ajplung.00452.2006.

108. Bello-Irizarry, S.N.; Wang, J.; Olsen, K.; Gigliotti, F.; Wright, T.W. The Alveolar Epithelial Cell Chemokine Response to Pneumocystis Requires Adaptor Molecule MyD88 and Interleukin-1 Receptor but Not Toll-Like Receptor 2 or 4. *Infection and Immunity* **2012**, *80*, 3912–3920, doi:doi:10.1128/iai.00708-12.

109. Bello-Irizarry, S.N.; Wang, J.; Johnston, C.J.; Gigliotti, F.; Wright, T.W. MyD88 Signaling Regulates Both Host Defense and Immunopathogenesis during Pneumocystis Infection. *The Journal of Immunology* **2014**, *192*, 282–292, doi:10.4049/jimmunol.1301431.

110. Pryhuber, G.S.; Huyck, H.L.; Bhagwat, S.; O'Reilly, M.A.; Finkelstein, J.N.; Gigliotti, F.; Wright, T.W. Parenchymal Cell TNF Receptors Contribute to Inflammatory Cell Recruitment and Respiratory Failure in Pneumocystis carinii-Induced Pneumonia1. *The Journal of Immunology* **2008**, *181*, 1409–1419, doi:10.4049/jimmunol.181.2.1409.

111. Wright, T.W.; Johnston, C.J.; Harmsen, A.G.; Finkelstein, J.N. Chemokine Gene Expression during <i>Pneumocystis carinii</i>-Driven Pulmonary Inflammation. *Infection and Immunity* **1999**, *67*, 3452–3460, doi:doi:10.1128/iai.67.7.3452-3460.1999.

112. Tasaka, S.; Kobayashi, S.; Kamata, H.; Kimizuka, Y.; Fujiwara, H.; Funatsu, Y.; Mizoguchi, K.; Ishii, M.; Takeuchi, T.; Hasegawa, N. Cytokine profiles of bronchoalveolar lavage fluid in patients with pneumocystis pneumonia. *Microbiol Immunol* **2010**, *54*, 425–433, doi:10.1111/j.1348-0421.2010.00229.x.

113. Chou, C.W.; Lin, F.C.; Tsai, H.C.; Chang, S.C. The importance of pro-inflammatory and anti-inflammatory cytokines in Pneumocystis jirovecii pneumonia. *Med Mycol* **2013**, *51*, 704–712, doi:10.3109/13693786.2013.772689.

114. Chou, C.-W.; Lin, F.-C.; Tsai, H.-C.; Chang, S.-C. The impact of concomitant pulmonary infection on immune dysregulation in Pneumocystis jirovecii pneumonia. *BMC Pulmonary Medicine* **2014**, *14*, 182, doi:10.1186/1471-2466-14-182.

115. Szymczak, W.A.; Deepe, G.S., Jr. The CCL7-CCL2-CCR2 axis regulates IL-4 production in lungs and fungal immunity. *J Immunol* **2009**, *183*, 1964–1974, doi:10.4049/jimmunol.0901316.

116. Locachevic, G.A.; Pereira, P.A.; Secatto, A.; Fontanari, C.; Galvão, A.F.; Prado, M.K.; Zoccal, K.F.; Petta, T.; Moraes, L.A.; Ramos, S.G.; et al. Erythropoietin Exacerbates Inflammation and Increases the Mortality of Histoplasma capsulatum-Infected Mice. *Mediators Inflamm* **2015**, *2015*, 786319, doi:10.1155/2015/786319.

117. Wüthrich, M.; Ersland, K.; Sullivan, T.; Galles, K.; Klein, Bruce S. Fungi Subvert Vaccine T Cell Priming at the Respiratory Mucosa by Preventing Chemokine-Induced Influx of Inflammatory Monocytes. *Immunity* **2012**, *36*, 680–692, doi:https://doi.org/10.1016/j.immuni.2012.02.015.

118. Souto, J.T.; Aliberti, J.C.; Campanelli, A.P.; Livonesi, M.C.; Maffei, C.M.; Ferreira, B.R.; Travassos, L.R.; Martinez, R.; Rossi, M.A.; Silva, J.S. Chemokine production and leukocyte recruitment to the lungs of Paracoccidioides brasiliensis-infected mice is modulated by interferon-gamma. *Am J Pathol* **2003**, *163*, 583–590, doi:10.1016/s0002-9440(10)63686-3.

119. Lopera, D.; Naranjo, T.W.; Cruz, O.G.; Restrepo, A.; Cano, L.E.; Lenzi, H.L. Structural and topographic dynamics of pulmonary histopathology and local cytokine profiles in Paracoccidioides brasiliensis conidia-infected mice. *PLoS Negl Trop Dis* **2011**, *5*, e1232, doi:10.1371/journal.pntd.0001232.

120. Costa, T.A.; Bazan, S.B.; Feriotti, C.; Araújo, E.F.; Bassi, Ê.; Loures, F.V.; Calich, V.L. In pulmonary paracoccidioidomycosis IL-10 deficiency leads to increased immunity and regressive infection without enhancing tissue pathology. *PLoS Negl Trop Dis* **2013**, *7*, e2512, doi:10.1371/journal.pntd.0002512.

121. Loures, F.V.; Pina, A.; Felonato, M.; Araújo, E.F.; Leite, K.R.M.; Calich, V.L.G. Toll-Like Receptor 4 Signaling Leads to Severe Fungal Infection Associated with Enhanced Proinflammatory Immunity and Impaired Expansion of Regulatory T Cells. *Infection and Immunity* **2010**, *78*, 1078–1088, doi:doi:10.1128/iai.01198-09.

122. Loures, F.V.; Pina, A.; Felonato, M.; Feriotti, C.; Araújo, E.F.d.; Calich, V.L.G. MyD88 Signaling Is Required for Efficient Innate and Adaptive Immune Responses to Paracoccidioides brasiliensis Infection. *Infection and Immunity* **2011**, *79*, 2470–2480, doi:doi:10.1128/iai.00375-10.
123. Pina, A.; Bernardino, S.; Calich, V.L.G. Alveolar macrophages from susceptible mice are more competent than those of resistant mice to control initial Paracoccidioides brasiliensis infection. *Journal of Leukocyte Biology* **2008**, *83*, 1088–1099, doi:10.1189/jlb.1107738.
124. Pedroso, V.S.P.; Vilela, M.C.; Santos, P.C.; Cisalpino, P.S.; Rachid, M.A.; Teixeira, A.L. Traffic of Leukocytes and Cytokine Up-regulation in the Central Nervous System in a Murine Model of Neuroparacoccidioidomycosis. *Mycopathologia* **2013**, *176*, 191–199, doi:10.1007/s11046-013-9679-3.
125. Lyon, A.C.; Teixeira, M.M.; Araújo, S.A.; Pereira, M.C.N.; Pedroso, E.R.P.; Teixeira, A.L. Serum levels of sTNF-R1, sTNF-R2 and CXCL9 correlate with disease activity in adult type paracoccidioidomycosis. *Acta Tropica* **2009**, *109*, 213–218, doi:https://doi.org/10.1016/j.actatropica.2008.11.010.
126. Zucker, K.E.; Kamberi, P.; Sobel, R.A.; Cloud, G.; Meli, D.N.; Clemons, K.V.; Stevens, D.A.; Williams, P.L.; Leib, S.L. Temporal expression of inflammatory mediators in brain basilar artery vasculitis and cerebrospinal fluid of rabbits with coccidioidal meningitis. *Clinical and Experimental Immunology* **2006**, *143*, 458–466, doi:10.1111/j.1365-2249.2006.03011.x.
127. Belic, S.; Page, L.; Lazariotou, M.; Waaga-Gasser, A.M.; Dragan, M.; Springer, J.; Loeffler, J.; Morton, C.O.; Einsele, H.; Ullmann, A.J.; et al. Comparative Analysis of Inflammatory Cytokine Release and Alveolar Epithelial Barrier Invasion in a Transwell® Bilayer Model of Mucormycosis. *Frontiers in Microbiology* **2019**, *Volume 9 - 2018*, doi:10.3389/fmicb.2018.03204.
128. Shete, A.; Deshpande, S.; Sawant, J.; Warthe, N.; Thakar, M.; Madkaikar, M.; Pradhan, V.; Rao, P.; Rohatgi, S.; Mukherjee, A.; et al. Higher proinflammatory responses possibly contributing to suppressed cytotoxicity in patients with COVID-19 associated mucormycosis. *Immunobiology* **2023**, *228*, 152384, doi:https://doi.org/10.1016/j.imbio.2023.152384.
129. Rosenblum Lichtenstein, J.H.; Molina, R.M.; Donaghey, T.C.; Brain, J.D. Strain differences influence murine pulmonary responses to Stachybotrys chartarum. *American journal of respiratory cell and molecular biology* **2006**, *35*, 415–423.
130. Leino, M.; Mäkelä, M.; Reijula, K.; Haahtela, T.; Mussalo-Rauhamaa, H.; Tuomi, T.; Hintikka, E.L.; Alenius, H. Intranasal exposure to a damp building mould, Stachybotrys chartarum, induces lung inflammation in mice by satratoxin-independent mechanisms. *Clin Exp Allergy* **2003**, *33*, 1603–1610, doi:10.1046/j.1365-2222.2003.01808.x.
131. He, M.; Ichinose, T.; Song, Y.; Yoshida, Y.; Kobayashi, F.; Maki, T.; Yoshida, S.; Takano, H.; Shibamoto, T.; Sun, G. The Role of Toll-Like Receptors and Myeloid Differentiation Factor 88 in Bjerkandera adusta-Induced Lung Inflammation. *Int Arch Allergy Immunol* **2015**, *168*, 96–106, doi:10.1159/000441895.
132. Trivedi, P.; Singh, S.; Dowari, S.; Jedhe, N.; Rana, K.; Srivastava, R.; Gajjar, D. Ex Vivo Goat Corneal Model for Investigating Host Immune Responses and Antifungal Drug Testing in Fusarium Keratitis. *Apmis* **2025**, *133*, e70120, doi:10.1111/apm.70120.
133. Tani, K.; Adachi, M.; Nakamura, Y.; Kano, R.; Makimura, K.; Hasegawa, A.; Kanda, N.; Watanabe, S. The effect of dermatophytes on cytokine production by human keratinocytes. *Archives of Dermatological Research* **2007**, *299*, 381–387, doi:10.1007/s00403-007-0780-7.
134. Selander, C.; Engblom, C.; Nilsson, G.; Scheynius, A.; Andersson, C.L. TLR2/MyD88-Dependent and -Independent Activation of Mast Cell IgE Responses by the Skin Commensal Yeast Malassezia sympodialis 1. *The Journal of Immunology* **2009**, *182*, 4208–4216, doi:10.4049/jimmunol.0800885.

135. Field, K.A.; Johnson, J.S.; Lilley, T.M.; Reeder, S.M.; Rogers, E.J.; Behr, M.J.; Reeder, D.M. The White-Nose Syndrome Transcriptome: Activation of Anti-fungal Host Responses in Wing Tissue of Hibernating Little Brown Myotis. *PLoS Pathog* **2015**, *11*, e1005168, doi:10.1371/journal.ppat.1005168.

136. Busch, R.; Murti, K.; Liu, J.; Patra, A.K.; Muhammad, K.; Knobeloch, K.P.; Lichtinger, M.; Bonifer, C.; Wörtge, S.; Waisman, A. NFATc1 releases BCL6-dependent repression of CCR2 agonist expression in peritoneal macrophages from Saccharomyces cerevisiae infected mice. *European journal of immunology* **2016**, *46*, 634–646.
